# Experimental Models of COVID-19

**DOI:** 10.3389/fcimb.2021.792584

**Published:** 2022-01-05

**Authors:** Luis A. Caldera-Crespo, Michael J. Paidas, Sabita Roy, Carl I. Schulman, Norma Sue Kenyon, Sylvia Daunert, Arumugam R. Jayakumar

**Affiliations:** ^1^ Department of Obstetrics, Gynecology and Reproductive Sciences, University of Miami Miller School of Medicine, Miami, FL, United States; ^2^ St. George’s University Graduate Medical Education Program, University Centre Grenada, West Indies, Grenada; ^3^ Department of Surgery, University of Miami Miller School of Medicine, Miami, FL, United States; ^4^ Department of Microbiology & Immunology, University of Miami Miller School of Medicine, Miami, FL, United States; ^5^ Department of Biomedical Engineering, University of Miami Miller School of Medicine, Miami, FL, United States; ^6^ Diabetes Research Institute, University of Miami Miller School of Medicine, Miami, FL, United States; ^7^ Department of Biochemistry and Molecular Biology, University of Miami Miller School of Medicine, Miami, FL, United States; ^8^ Dr. JT Macdonald Foundation Biomedical Nanotechnology Institute, University of Miami Miller School of Medicine, Miami, FL, United States; ^9^ University of Miami Clinical and Translational Science Institute, University of Miami Miller School of Medicine, Miami, FL, United States

**Keywords:** SARS-CoV-2, COVID-19, experimental models of COVID-19, pathology, variants of concern, MHV-1, pneumonia, *in vitro* model

## Abstract

COVID-19 is the most consequential pandemic of the 21^st^ century. Since the earliest stage of the 2019-2020 epidemic, animal models have been useful in understanding the etiopathogenesis of SARS-CoV-2 infection and rapid development of vaccines/drugs to prevent, treat or eradicate SARS-CoV-2 infection. Early SARS-CoV-1 research using immortalized *in-vitro* cell lines have aided in understanding different cells and receptors needed for SARS-CoV-2 infection and, due to their ability to be easily manipulated, continue to broaden our understanding of COVID-19 disease in *in-vivo* models. The scientific community determined animal models as the most useful models which could demonstrate viral infection, replication, transmission, and spectrum of illness as seen in human populations. Until now, there have not been well-described animal models of SARS-CoV-2 infection although transgenic mouse models (i.e. mice with humanized ACE2 receptors with humanized receptors) have been proposed. Additionally, there are only limited facilities (Biosafety level 3 laboratories) available to contribute research to aid in eventually exterminating SARS-CoV-2 infection around the world. This review summarizes the most successful animal models of SARS-CoV-2 infection including studies in Non-Human Primates (NHPs) which were found to be susceptible to infection and transmitted the virus similarly to humans (e.g., Rhesus macaques, Cynomolgus, and African Green Monkeys), and animal models that do not require Biosafety level 3 laboratories (e.g., Mouse Hepatitis Virus models of COVID-19, Ferret model, Syrian Hamster model). Balancing safety, mimicking human COVID-19 and robustness of the animal model, the Murine Hepatitis Virus-1 Murine model currently represents the most optimal model for SARS-CoV-2/COVID19 research. Exploring future animal models will aid researchers/scientists in discovering the mechanisms of SARS-CoV-2 infection and in identifying therapies to prevent or treat COVID-19.

## 1 Introduction

Severe acute respiratory syndrome coronavirus 2 (SARS-CoV-2), also referred to as human coronavirus 19 (HCoV-19), is the virus that causes coronavirus disease (COVID-19). SARS-CoV-2 is believed to be a respiratory virus although it exerts a severe impact on other organs resulting in multi-organ failure (Mokhtari et al., 2020; Zaim et al., 2020; Farooqui, 2021; Loganathan et al., 2021; Paidas et al., 2021);. SARS-CoV-2 is a positive single stranded RNA virus, with a single linear RNA segment that causes more severe disease than any other coronavirus. The US National Institutes of Health described it as the successor to SARS-CoV-1, the virus that caused the SARS 2002 outbreak. SARS-CoV-2 was first identified in the city of Wuhan, Hubei, China. The World Health Organization declared the outbreak a Public Health Emergency of International Concern on 30^th^ January, 2020 and SARS-CoV-2 was subsequently declared as a pandemic on 11^th^ March 2020.

SARS-CoV-2 is an airborne virus that infects its host by first binding respiratory epithelium in the upper airways. At this time, the host may complain of non-specific flu-like symptoms (i.e. fever, fatigue, rhinorrhea). However, it is common for hosts to remain asymptomatic during an extended incubation period of 5-14 days (Hu et al., 2021). During the incubation period, asymptomatic transmission is possible and the virus itself has been shown to be highly infectious in close quarters and poorly ventilated areas. Within the host, the virus then migrates to the lower airways where it binds to a receptor of a membrane protein that regulates the renin-angiotensin system [(angiotensin converting enzyme 2 (ACE-2)], allowing viral uptake within the cell; systemic infection then ensues (Hu et al., 2021). The host’s own cellular-mediated immune response most likely is responsible for the severe illness that patients can experience when infected (Wang et al., 2020). Most characteristic of the SARS-CoV-2 virus is the atypical “walking” pneumonia, causing patients to appear healthy & lucid at their baseline health while at the same time harbor dangerously low oxygen saturation levels in the severely hypoxic range, emphasizing the importance of diagnostic imaging such as CT in aiding clinical diagnosis (Larici et al., 2020).

Due to the virus’s specific tropism with host interaction, there are also symptoms that can help differentiate from the common cold or influenza, such as neurological symptoms (i.e. anosmia, encephalopathy) and GI symptoms (i.e. diarrhea, weight loss) (Huang et al., 2020). The most severely infected patients may present to emergency departments and indicate treatment for Acute Respiratory Distress Syndrome (ARDS) (Qiu et al., 2020). Current treatment protocols for moderate and severe disease are based upon success with steroids, laying the patient prone, and other therapies (Malin et al., 2020; Sood et al., 2020) (i.e. Remdesivir). Vaccine development has been the most crucial development in COVID-19 prophylaxis, especially in the highly exposed (i.e. emergency rooms) as well as the most vulnerable populations (i.e. nursing homes).

There has been debate as to whether proximity to an infected person, duration of exposure, or viral load is the primary determinant for risk of infection. Comorbidities, advanced age, and immunosuppression were the highest risk factors for severe illness from COVID-19. However, the virus has also caused severe disease in seemingly healthy younger populations and caused lasting effects which continue to debilitate patients long after disease remission, hence long COVID (Yong, 2021). Studies to understand the epidemiology of SARS-CoV-2 infection in children have been limited (Rankin et al., 2021). Children have been shown to be able to transmit the virus at much lower rates than adolescents, but with no data suggesting increased susceptibility to disease (Meyer et al., 2021). However, there is debate on whether children are able to transmit the virus asymptomatically, which could affect household transmission. Conversations in the public sector regarding in-person classroom settings are hotly debated in today’s government halls and public forums.

By comparing the DNA to prior coronaviruses (i.e. SARS-CoV-1 & MERS), SARS-CoV-2 has been shown to be most similar in structure and transmission to the SARS-CoV-1 virus. The virus most likely originated in bat hosts, and may or may not have infected an intermediate host, such as pangolins (Goh et al., 2020). Regardless of SARS CoV-2’s exact infectious route, human cells have shown to be highly tropic to the virus, specifically epithelial cells with human ACE-2 receptors, which line organs such as respiratory and GI tracts. Understanding how the virus infects its hosts is crucial in the production of vaccines that can help prevent disease (Chaudhari et al., 2021).

### 1.1 Variants of SARS-CoV-2

Many variants of SARS-CoV-2 have been identified, which are grouped into the much larger clades. Different clade nomenclatures have been proposed. Nextstrain divides the variants into five clades (19A, 19B, 20A, 20B, and 20C), while GISAID divides them into seven (L, O, V, S, G, GH, and GR). Several notable variants of SARS-CoV-2 emerged in late 2020. The World Health Organization has currently declared four variants of concern, which are as follows:

**Alpha**: Lineage B.1.1.7, found to be derived from the SARS-CoV-2 20B/GR clade, emerged in the United Kingdom in September 2020, with evidence of increased transmissibility and virulence. Notable mutations at the viral S gene include N501Y, an asparagine to tyrosine amino acid substitution at position 501, and P681H, a proline to histidine substitution at position 681. An E484K mutation, glutamate to lysine substitution at position 484 of the RBD, in some lineage B.1.1.7 virions has been noted and also tracked by various public health agencies (Tang et al., 2021). Researchers are currently investigating the etiology of lineage B.1.1.7 and hypothesize that these mutations may have occurred due to prolonged infection of an immunocompromised host (Choi et al., 2020). Such mutations, especially the E484K arising in South Africa, are concerning for their possible gain-of-function ability to confer advantage against current convalescent plasma treatments as well as vaccines.

**Beta**: Lineage B.1.351 cases were first reported in South Africa on December 2020 (Hoffmann et al., 2021), with evidence of increased transmissibility and changes to antigenicity, with some public health officials raising alarms about its impact on the efficacy of some vaccines. Notable mutations include K417N, E484K and N501Y, with 12 total mutations and one deletion compared to the original Wuhan strain, and most mutations in the Spike protein domain, causing concern for a possible gain-of-function escape from neutralizing antibodies (Rees-Spear et al., 2021). There have been promising studies making use of monoclonal antibodies that may serve as therapeutics in treating mutated variants of concern (Du et al., 2021).

**Gamma**: Lineage P.1, or B.1.1.28.1, emerged in Brazil in November 2020, also with evidence of increased transmissibility and virulence, alongside changes to antigenicity. Similar concerns about vaccine efficacy have been raised due to P.1’s high number of accumulated mutations in the S protein which is the suggested cause for a rapid increase in hospital admissions. Notable mutations also include K417N, E484K and N501Y, a group of mutations that are highly suspected for variants ability to escape antibody-mediated immunity (Rees-Spear et al., 2021).

**Figure 1:**
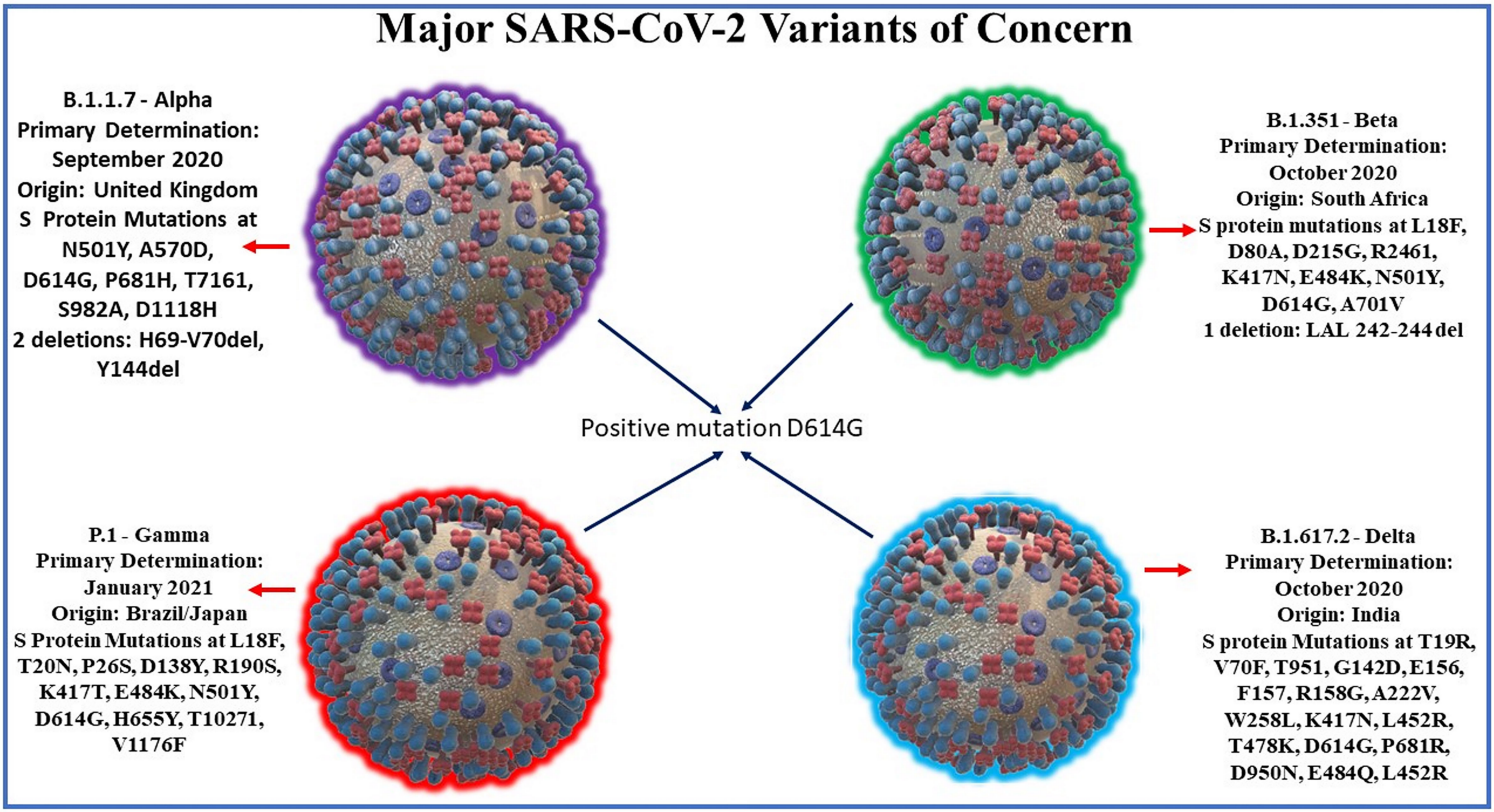
Major SARS-CoV-2 variants along with their dates and location of primary determination and their characteristic spike protein mutation sites. SARS-CoV-2 variants share a common mutation in D614G of the spike protein. Notable spike mutations: Alpha variant mutations include N501Y, an asparagine to tyrosine amino acid substitution at position 501, and P681H, a proline to histidine substitution at position 681have been found to be preserved in subsequent variants. The Beta variant E484K mutation, glutamate to lysine substitution at position 484 of the RBD arising from South Africa may lead to possible gain-of-function ability to confer advantage against current convalescent plasma treatments. Beta & Gamma (Brazil/Japan) lineage mutations also include K417N and N501Y and may together lead to possible gain-of-function escape from neutralizing antibodies. Delta variant, first discovered in India 2020, mutations include L452R substitution correlates with higher affinity to the ACE2 host receptor, & P681R may be responsible for increased infectivity of host cells.

**Delta**: Lineage B.1.617.2 emerged in India on October 2020. There is also evidence of increased transmissibility and changes to antigenicity due to mutations in the gene encoding the SARS-CoV-2 Spike protein. Of the 17 mutations found in the Delta genome, four are of major concern: D614G substitution is also found in other highly transmissible variants, T478K substitution, L452R substitution correlates with higher affinity to the ACE2 host receptor, & P681R may be responsible for increased infectivity of host cells (Lazarevic et al., 2021; Lustig et al., 2021). A recent cohort study in England found increased hospital admission or emergency care in patients infected with delta variant compared to those infected with alpha variant, suggesting that outbreaks of delta variant in unvaccinated patients may lead to higher burdens on healthcare systems compared to alpha variant cases (Twohig et al., 2021). Other notable variants include 6 other WHO-designated variants under investigation and Cluster 5, which emerged among mink in Denmark and resulted in a mink euthanasia campaign rendering it virtually extinct (Larsen, 2021). See **Figure 1** for SARS-CoV-2 variants and its mutation points.

More recently discovered variants include Lambda (C.37) - a variant of interest first reported to WHO from Peru on August 2020, Mu (B.1.621) - a variant of interest first reported to WHO from Colombia on January 2021, and Omicron (B.1.1.529) - a variant of concern first reported to WHO from South Africa and Botswana on November 2021. Additionally, there are a number of variants under monitoring by the CDC. These include C.36+L452R, B.1.1.318, P.1+P681H, B.1.617.2+K417N, C.1.2, B.1.617.2+E484X, B.1.617.2+Q613H, B.1.617.2+Q677H, and B.1.640. Based on the mode of transmission and rates of worldwide spread, new variants will continue to emerge.

**Figure 2:**
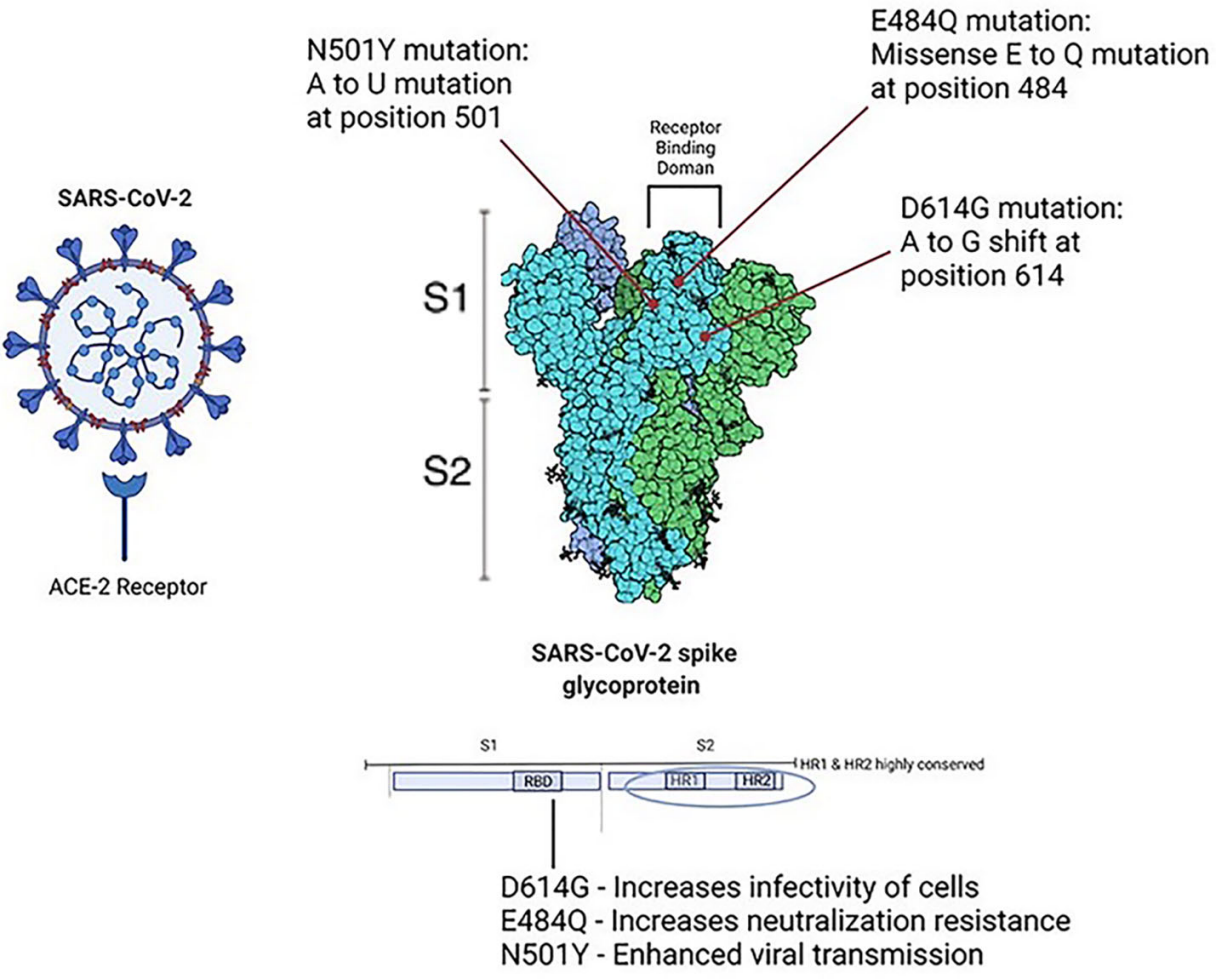
The structural and functional aspects of SARS-CoV-2. Point mutations in Spike protein have been shown to increase survival of SARS-CoV-2 virion from host immune response. D614G is related to increased infectivity of cells, E484Q has been shown to increase neutralization resistance, and N501 Y has been shown to cause enhanced viral transmission.

*Viral structure and predictions*: Phylogenetic analyses of SARS-CoV-2 variants shed light on the mutations responsible for more infectious strains. The structure of the virion can be determined through analysis of its coding regions. These include basic components which comprise coronaviridae: Spike protein (S), Membrane protein (M), Envelope protein (E), Nucleoprotein (N), and Ribonucleic Acid (RNA). The Receptor Binding Domain (RBD) found on Spike protein residues is responsible for binding of the ACE-2 receptor, allowing the virion cell entry into its host. Further, Hoffman et al. (2020) reported that SARS-CoV-2 cell entry was dependent on the presence of both the ACE2 receptor and a serine protease TMPRSS2. Choi et al. (2020) conducted phylogenetic analyses in an immunocompromised patient who succumbed to persistent infection and found that viral evolution was found to be rooted in amino acid changes predominantly in the spike gene and receptor-binding domain. A small portion of the entire viral genome was responsible for the majority of observed changes in viral persistence. The spike protein itself can further be divided into its two subunits: 1) S1 subunit, the globular receptor binding domain containing the receptor binding motif, responsible for binding to the host cell receptor; 2) S2 subunit, the stalk fusion domain, responsible for the fusion of viral and cellular membranes (Mittal et al., 2020). Studies have shown that the S1 subunit of the RBD has 10 to 20 fold higher affinity for the ACE-2 receptor when compared to SARS-CoV-1 RBD (Wrapp et al., 2020). The HR1 and HR2 domains which comprise the S2 subunit have been found to be highly stable, further suggesting the importance of the Spike protein in the infectivity of SARS-CoV-2 (Xia et al., 2020). SARS-CoV-2 entry requires cleavage of the Spike protein at the S1/S2 cleavage site, and this cleavage is carried out by furin protease (Peacock et al., 2021). The P681H mutation at the PRRAR furin cleavage site, a polybasic insertion provides a selective advantage to SARS-CoV-2 in human airway epithelial cells, allowing more favorable binding of furin to the S protein, and thus enhanced membrane fusion (Mohammad et al., 2021). See **Figure 2** for structural and functional aspects of SARS-CoV-2 spike protein. Recent studies have shown/suggested other possible receptors for SARS-CoV-2. These include 1) a transmembrane glycoprotein CD147 and a receptor on host cells commonly known as basic immunoglobulin (Basigin) or extracellular matrix metalloproteinase inducer (EMMPRIN), 2) a transmembrane protein Neuropilin-1 (NRP-1), 3) an ectopeptidase dipeptidyl peptidase 4 (DPP4) also known as CD26, 4) alanyl aminopeptidase (ANPEP), 5) glutamyl aminopeptidase (ENPEP), and 6) angiotensin II receptor type 2 (AGTR2) (see Masre et al., 2021 and references therein). Further, studies have also identified and explored intersection genes of niacin such as Bcl-2-like 1 (BCL2L1), prostaglandin-endoperoxide synthase 2 (PTGS2), interlukin-1-β, interferon gamma, plasminogen activator inhibitor-1 (SERPINE1) and COVID-19 (Li et al., 2021), probably involved in viral spread and can be targeted for potential therapy.

## 2 Animal Models of COVID 19

Important elements in vaccine production and drug testing include the selection of animal models based upon specific criteria (Davidson et al., 1987): They must be able to: 1) replicate life cycle of the pathogen (i.e. incubation period similar to humans); 2) Demonstrate similar symptoms as seen in humans when infected with SARS-CoV-2; 3) recapitulate the illness brought on by physiological viral dosing as seen in humans. (It would not be useful for animal models to only exhibit infection when given a dose unlikely to be seen clinically); 4) transmit the viral pathogen in same manner as in humans (i.e. virus can infect respiratory tract of animal model); and 5) measure the immune response of the animal model (i.e. acute phase reactants vs seroconversion); 6) enable low cost and highly practical models which allow for models and results to be easily reproduced in a lab setting; and 7) provide acceptable ecological as well as ethical consequences during experimentation.

Laboratory mice/rats are the most commonly used animals in preclinical research, as well as the most useful and appropriate resource for mechanistic investigations. Additionally, they are easy to handle, and their size, and high reproduction number further assist in identifying the therapeutic potential of drugs. The SARS-CoV-2 spike protein requires the human ACE2 receptor (hACE2) for cellular entry and infection (Wang et al., 2020). Wan et al. demonstrated that exposure of wild-type mice to SARS-CoV-2 lack the ability to be infected due to differences in their ACE2 receptors (Wan et al., 2020). Mouse models were genetically engineered to express hACE2 by manipulating different promoter regions and were successfully infected with SARS CoV-2, making them useful for vaccine and therapeutic research. A disadvantage of these mouse models includes their limited availability.

### 2.1 K18-hACE2 Transgenic Mouse Model

Transgenic expression of hACE2 in mice allows for infection following exposure to SARS- CoV-2. Winkler et al. (2020) were able to demonstrate that mice genetically engineered to express epithelial cell cytokeratin-18 promoter (k-18) hACE2 gene, originally used in SARS research, were able to be infected similarly to human hosts (Winkler et al., 2020). Expression of this gene allowed hACE2 to be expressed in multiple tissues after mice were inoculated with SARS-CoV-2 in the intranasal epithelial passages, similar to SARS-CoV-2 tropism in human respiratory passages. Viral RNA was detected consistently in these tissues and showed pathological evidence of severe infection which allowed researchers to compare their findings with human subjects. Winkler noted that while this model is useful in studying severe SARS-CoV-2 infection in humans, some limitations of this model included the risk of ectopic hACE2 expression which changes the cellular tropism of the virus. Another limitation is that hACE2 is expressed at non-physiological levels due to unique k18 promoter in this model. Furthermore, this model does not take into account the complex interplay of hACE2 expression that can be seen in humans with certain comorbidities such as hypertension, cardiovascular disease and diabetes, as well as obesity, COPD & liver disease; diseases that have been shown to increase susceptibility to SARS-CoV-2 infection and increase the risk for development of COVID-19. (Ejaz et al., 2020).

Seibert et al. (2021) made use of the k18-hACE2 model to better understand the effect of SARS-CoV-2 infection on lung and GI microbiome diversity, an important factor in the immune system of infected hosts. Additionally, Liu et al. (2021) used this model when they reverse-engineered SARS-CoV-2 clones that could be used to further understand viral pathogenesis, as well as COVID-19 variants. Arce and Costoya (2021) further investigated the usefulness and limitations of the k18-hACE2 model and found that extrapulmonary infection symptoms manifest in a mild manner due to the high concentration of ACE2 receptors in lung tissue, and thus would not be useful in understanding the severe clinical symptoms that can be seen in COVID-19 patients.

k18-hACE2 transgenic mice have also been used in research of the pathogenesis of SARS-CoV-2 infection (Zheng et al., 2021). This study reproduced the histopathological lung disease, in a dose-dependent manner, as was seen in humans. These authors further demonstrated SARS-CoV-2’s ability to replicate in the sinonasal epithelium of the k18-hACE2 model and also supporting cells of olfactory neurons (not the olfactory neurons themselves), causing anosmia which was a common clinical symptom reported in humans associated with SARS-CoV-2 infection (seen more in females than males). This study also investigated the usefulness of convalescent plasma from COVID-19 patients in preventing mortality and clinical symptoms at certain levels of inoculum (<10^5^) (Zheng et al., 2021). However, as noted above, the limitation in the use of transgenic mouse models to study COVID-19 pathogenesis is that there is a risk of ectopic hACE2 expression, causing a change in the cellular tropism of the virus and thus a decrease in the usefulness of the model itself.

### 2.2 Mouse ACE2 Promoter With Human ACE2 Coding Sequence

Bao et al. (2020) were able to demonstrate the pathogenesis of SARS CoV-2 in transgenic mice [adult/aged male and female mice (6-11 month old)] which expressed hACE2 by way of the mouse ACE2 promoter. This model was produced by injection of mouse ACE2 promoter linked with human ACE2 coding gene into pronuclei of ICR mice fertilized ova. Bao found that ACE2 was mostly expressed in lungs, GI tract (intestines), kidneys and heart. Both wild-type and hACE2 transgenic mice were then inoculated with SARS-CoV-2 HB01 strain at 50% tissue culture infected dose (TCID50) and found weight changes, clinical symptoms, and death. The mice were then dissected at differing days post-infection so as to compare the histopathological changes in infected tissues (Bao et al., 2020). Limitations noted in this model were lack of symptoms which would not be useful in the study of clinical symptoms.

### 2.3 Endogenous Mouse ACE2 Promoter Model

One of the strategies scientists have employed to increase the specificity of the SARS-CoV-2 spike protein to endogenous mACE2 is to perform sequential passage of the virus in the animal model, thus over time causing an increase in viral tropism for the receptor, which results in infection and mortality in these mice, as seen in humans. Zhang et al. (2021) were able to demonstrate the usefulness of this model. Both aged (12 months) and young (2 months) mice were infected intranasally with 1 x 10^5^ of TCID50 of SARS-CoV-2 and collected samples of tissue & blood 3, 5, and 7 days post infection. Samples were analyzed for evidence of infection and viral replication. Notable findings of the study were the aged BALB/c model’s ability to support viral replication in airways (lungs, trachea and nasal epithelium), and develop interstitial pneumonia, and neutralizing antibodies. These authors further demonstrated a rapid adaptation of SARS-CoV-2 to aged BALB/c mice (Zhang et al., 2021), the delayed viral clearance seen in older populations, and shed light on the immune response to infection. This study also noted the model’s usefulness in creating mouse-adapted strains, as well as aiding in understanding of COVID-19 disease in older human populations. Limitations in this study included mice with only mild clinical symptoms compared to transgenic models.

**Figure 3:**
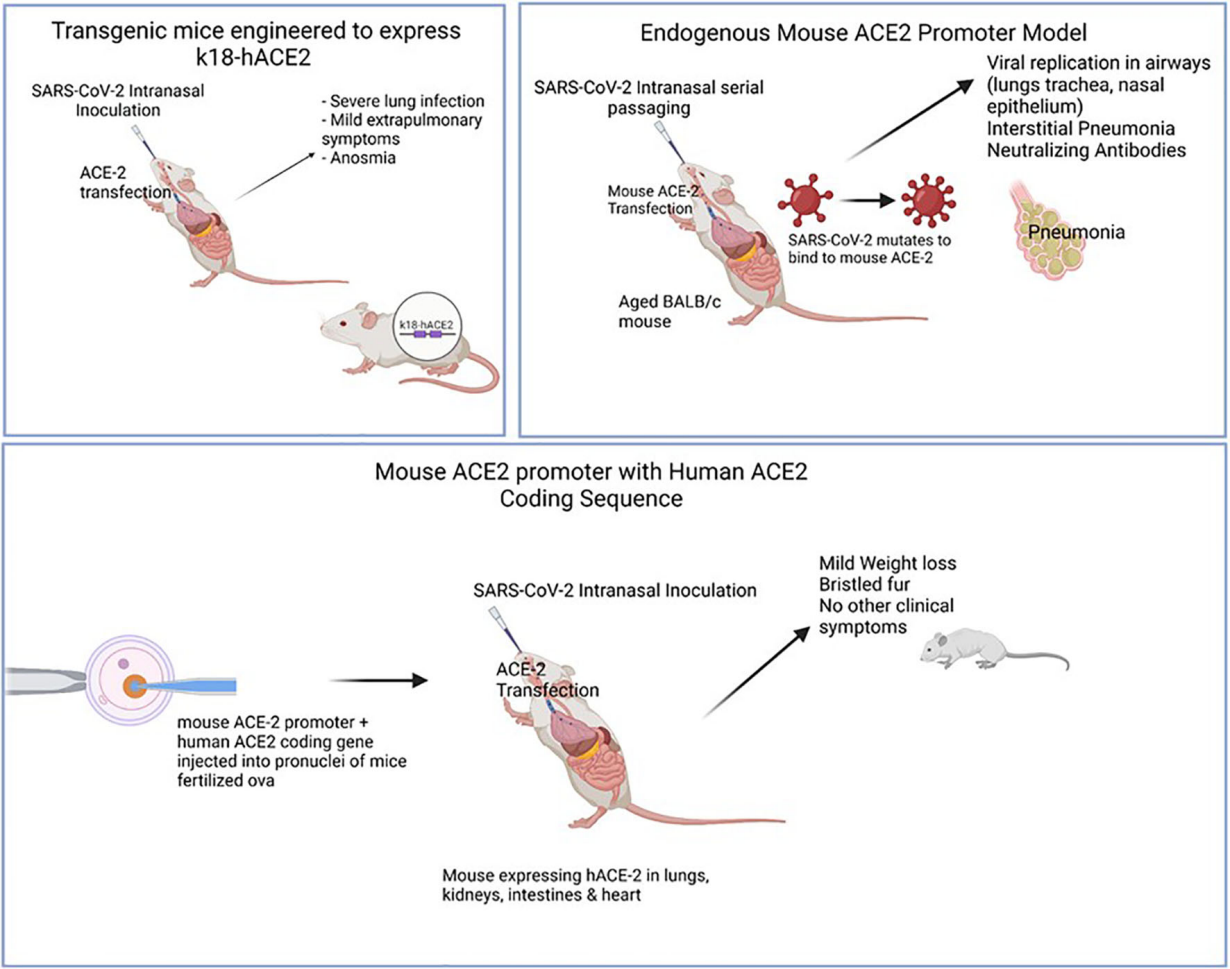
Illustrates various animal models of COVID-19 and the mode of infection. Models were useful in displaying different levels of clinical disease severity once sensitized to SARS-CoV-2 infection. Top left: Mice engineered to express hACE2 gene using k18 promoter. SARS-CoV-2 intranasal inoculation & ACE-2 transfection was shown to cause clinical symptoms (severe lung infection, anosmia) with mild extrapulmonary symptoms (GI, neurological). Top right: Aged, wild-type mice were serially passaged with intranasal SARS-CoV-2. Over time, viral generations eventually were able to bind to mACE-2 leading to clinical interstitial pneumonia and positive titers of neutralizing antibodies. Bottom: Mouse ACE-2 promoter with Human ACE-2 coding sequence were injected into pronuclei leading to mACE-2/hACE-2 susceptible to SARS-CoV-2 infection. hACE-2 receptors were expressed in lungs, kidneys, intestines & heart with mild clinical symptoms (mild weight loss, bristled fur).

Huang et al. (2021) were able to use the endogenous mouse model in their study in which they serially passaged SARS-CoV-2 until a mouse–adapted strain was obtained. Using lab analyses (complete deep gene sequencing, indirect immunofluorescence analysis, and interferometry), the authors were able to demonstrate that two mutations, Q493K and Q98H, in the RBD of the Spike protein of a mouse-adapted strain of SARS-CoV-2 (WBP-1) resulted in an increased affinity for mouse ACE2, causing increased infectivity and severe COVID-19. They were also able to use this model to demonstrate the effectiveness of a TLR7/8 agonist at protecting mice against WBP-1; a useful finding in understanding the pathogenesis of variant strains, as well as mechanisms of possible therapies (Huang et al., 2021). See **Figure 3** for comparison of animal models.

### 2.4 Adenovirus hACE2 Mouse Model for SARS-CoV-2 Infection

Hassan et al. (2020) developed the Adenovirus Human-ACE2 Mouse Model (Adv-hACE2) for SARS-CoV-2 infection by demonstrating the effectiveness of transduction in inducing the expression of hACE2 in mice. Young and adult mice (between 3-4 weeks old or 8-10 weeks old) were used in this study. Mice infected intranasally with SARS-CoV-2 (2.5 x10^8^ PFU) in the Adenovirus hACE2 Model showed a greater weight loss, clinical symptoms, and pathology in lung sections in addition to the absence of IFN signaling. While further investigation is needed, these findings suggest the possible protective effects of IFN signaling against viral SARS-CoV-2 infection.

**Figure 4:**
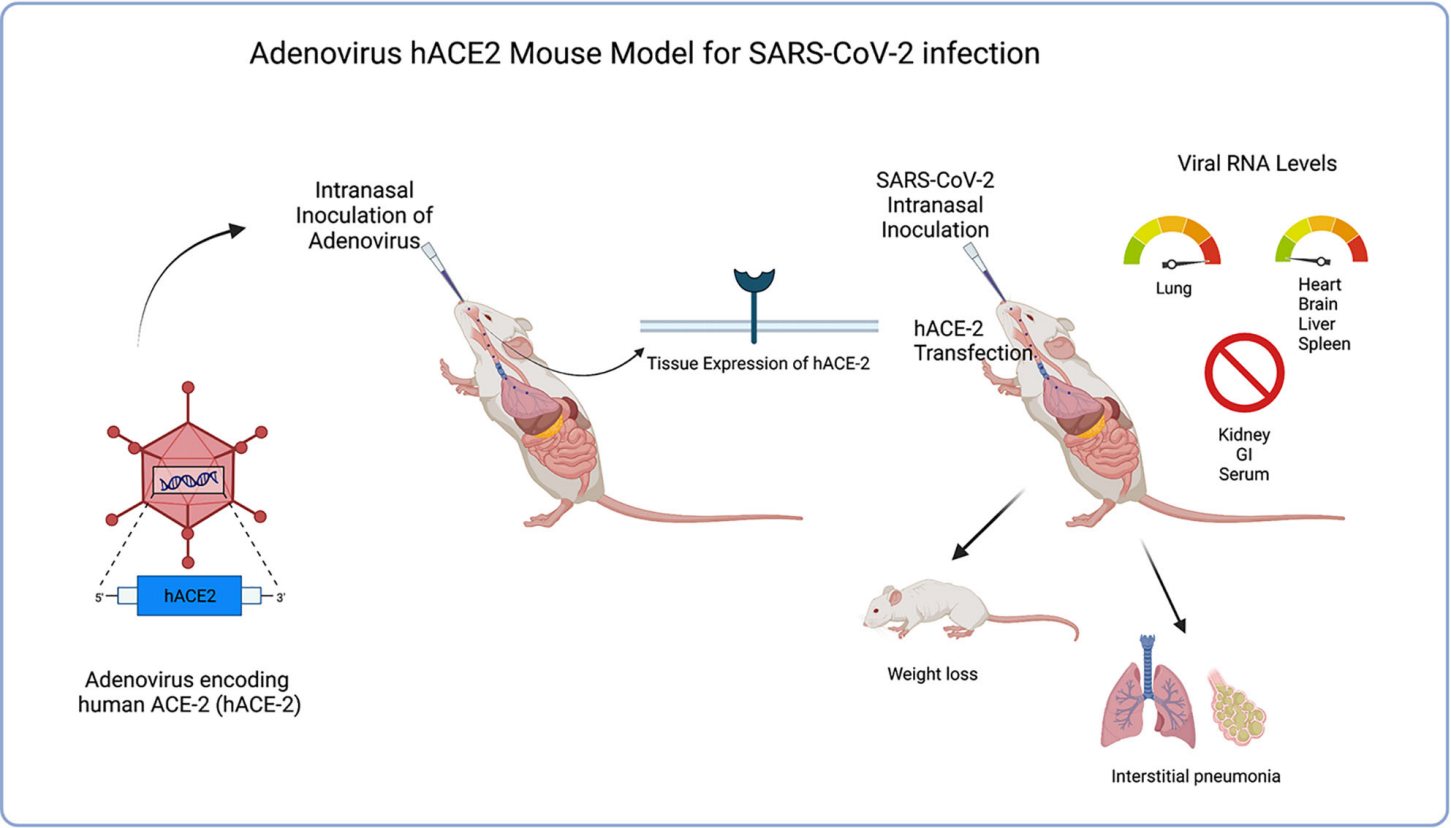
Illustrates the process of Adenovirus hACE2 mouse model transgenesis as well as clinical symptoms of model after SARS-CoV-2 inoculation. Adv-hACE-2 mice showed greater weight loss, clinical symptoms (interstitial pneumonia, weight loss), and pathology in lung sections in addition to the absence of IFN signaling. Elevated viral RNA levels are seen in lung tissue, but low levels are shown in other tissues (heart, brain, liver spleen) while other tissues are not tropic (kidney, GI, serum). Benefits of this model include the ability to quickly sensitize different species of mice due to the rapid deployability of the adenovirus delivery system for expressing hACE2.

It was concluded that benefits to this model would be the ability to quickly sensitize different species of mice due to the rapid deployability of the adenovirus delivery system for expressing hACE2 in mice, thus potentially accelerating the pace of vaccines and therapeutic drug developments, as well as advancing to NHP and human trials. The costly and time-consuming procedure of genetically engineering transgenic mice to express hACE-2 could greatly be reduced by simply infecting wild-type lab mice with an Adenovirus vector leading to expression of hACE-2 and thus increasing susceptibility to SARS-CoV-2 infection. Han et al. (2021) further supported the Adv-hACE2 model in SARS-CoV-2 research by independently demonstrating its ability to be rapidly deployed in all transgenic and wild-type mice, proving Adv-hACE2 to be a beneficial model in testing vaccine candidates and therapies. More recently, Rai et al. (2021) used the Adv-hACE2 model in SARS-CoV-2 research to study the effects of obesity on COVID-19 disease severity and noted significant changes in cytokine expression in infected obese mice compared to lean mice, further proving the relative usefulness of this model in current COVID-19 research, Noted limitations would be differences in concentration of hACE2 expression in tissues from mouse-to-mouse, local and temporally-limited induced receptor expression, as well as inflammation of airways with Adv inoculation. Further limitations noted in this study were the induction of receptor density localized in lungs/low number in extrapulmonary organs making this Adv model less helpful in studies to treat extrapulmonary COVID-19 manifestations. See **Figure 4**.

### 2.5 Mouse Hepatitis Virus Model

Murine hepatitis virus strains have been shown to produce a clinically relevant model of severe acute respiratory syndrome in mice. The ability of a large number of murine coronavirus strains, including MHV-1, MHV-3, MHV-A59, MHV-JHM, and MHV-S, has been tested in various mice strains (from 6-8 week old Female BALB/cJ, C57BL/6J A/J, and C3H/St mice) for whether these viruses produce SARS-like pathology.

#### 2.5.1 MHV-1 Virus

**Figure 5:**
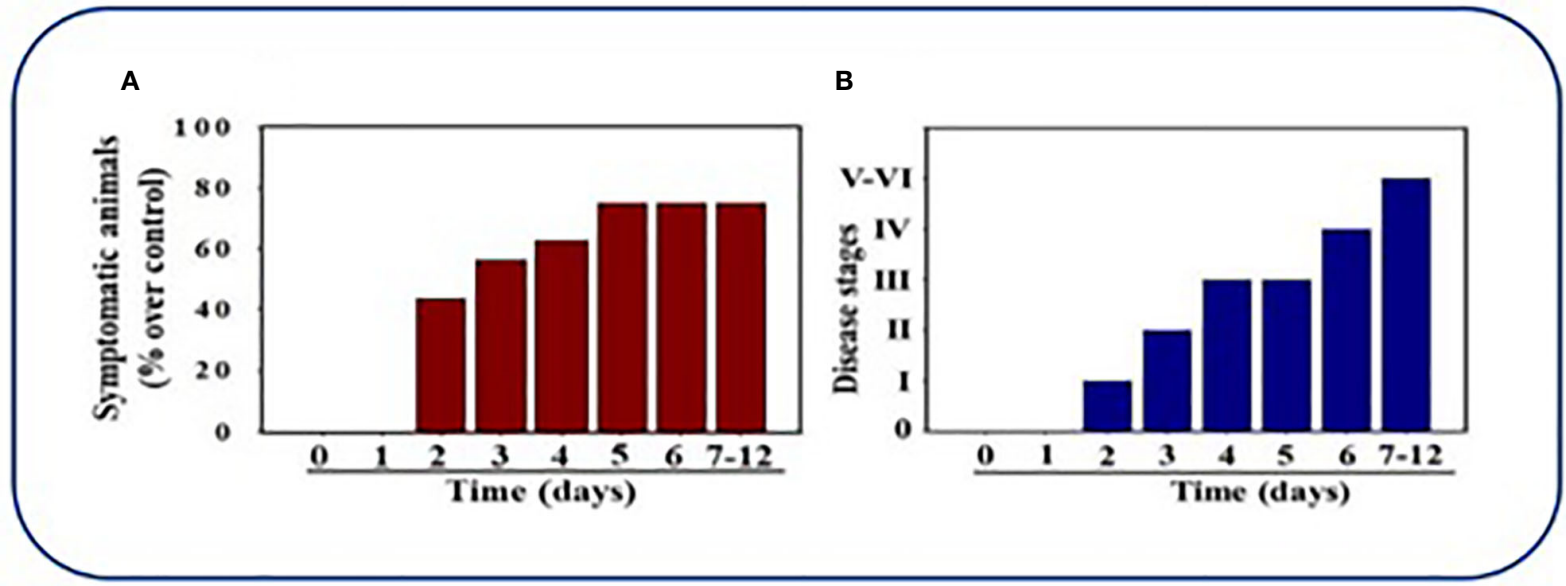
Mice infected by MHV-1 inoculation. **(A)** 40% of mice were symptomatic by Day 2 post-infection and exhibition of clinical signs progressively increased to 75%. **(B)** Mice inoculated by MHV-1 exhibited clinical signs starting at 2 days post-infection. Mild to moderate stage (I-III) of clinical symptoms were seen on days 2-4. Severe sickness (IV-VI) was seen in mice after 6 days. Reproduced with permission from Paidas et al, Viruses; Published by MDPI, 2021.

De Albuquerque et al. (2006) were able to produce clinical symptoms and pathology with the MHV-1 strain in their research in further understanding SARS and concluded in their studies that A/J mice were highly susceptible to MHV-1 infection and provided a useful model in human SARS so as to understand pathogenesis and for use in treatment innovation. Paidas et al. (2021) were recently published for data gathered which provide support for the MHV-1 model as a clinically important model for use in COVID-19 research. MHV-1 infected female mice were observed post-inoculation. These mice were then euthanized and their tissue histopathology were examined. Six different disease stages based on clinical progression of symptoms were established prior to inoculation to aid in observation of the infected models. 40% of inoculated mice exhibited clinical symptoms by day 2, with the number of symptomatic mice increasing until up to 75% of mice had signs of clinical disease by 7-12 days. The majority of MHV-1-infected mice initially developed only mild pulmonary disease (Stage I: drowsiness + lack of movement) similar to that seen in BALB/cJ mice by day 2 post-infection. These mice progressed to Stage II symptoms (ruffled fur + altered hind limb posture) noted on day 3 post-inoculation and further to Stage III (ruffled fur + mildly labored breathing) by day 4 & 5. In the majority of infected mice, pulmonary disease progressed to Stage IV (ruffled fur + inactivity + moderately labored breathing + tremor) on day 6, and by days 7-12 Stage V and VI of clinical disease were noted in the infected mice (ruffled fur + obviously labored breathing and lethargy; moribund state + death). See **Figures 5A, B**.

Histopathological examination of MHV-1-infected mice at day 7 showed inflammation (i.e. granular degeneration of cells, and migration of leukocytes into the lungs), along with proteinaceous debris filling the alveolar spaces with fibrillar to granular eosinophilic protein strands caused by progressive breakdown of the capillary wall and epithelial integrity, which permits leakage of protein rich edematous fluid into the alveoli, and the presence of hemosiderin-laden macrophages (indicating pulmonary congestion with dilated capillaries and leakage of blood into alveolar spaces). Furthermore, peribronchiolar interstitial infiltration, bronchiole epithelial cell necrosis and necrotic cell debris within alveolar lumens, alveolar exudation, hyaline membrane formation and alveolar hemorrhage with red blood cells within the alveolar space and interstitial edema are all characteristic features of infected lungs observed in humans with SARS-CoV-2 infection (Paidas et al., 2021).

Examination of the livers of MHV-1-infected A/J mice showed near normal histology to day 6, but on day 7 just prior to death, there was severe hepatic congestion, hepatocyte degeneration, severe periportal hepatocellular necrosis with pyknotic nuclei, ballooned hepatocytes, vacuolation, presence of piecemeal necrosis, as well as hemorrhagic changes. Ground glass hepatocytes show voluminous, abundant, granular cytoplasm, with peripheral cytoplasmic clearing and central nuclei, and apoptotic (acidophil) bodies, as well as absent hepatocytes replaced by abundant inflammatory cells. Condensation and dark staining of the cytoplasm and absence of the nucleus, fatty changes, binucleated hepatocytes, and activated Kupffer cells were also observed in MHV-1 exposed mice livers, compatible with lung and heart failure as described in humans.

**Figure 6:**
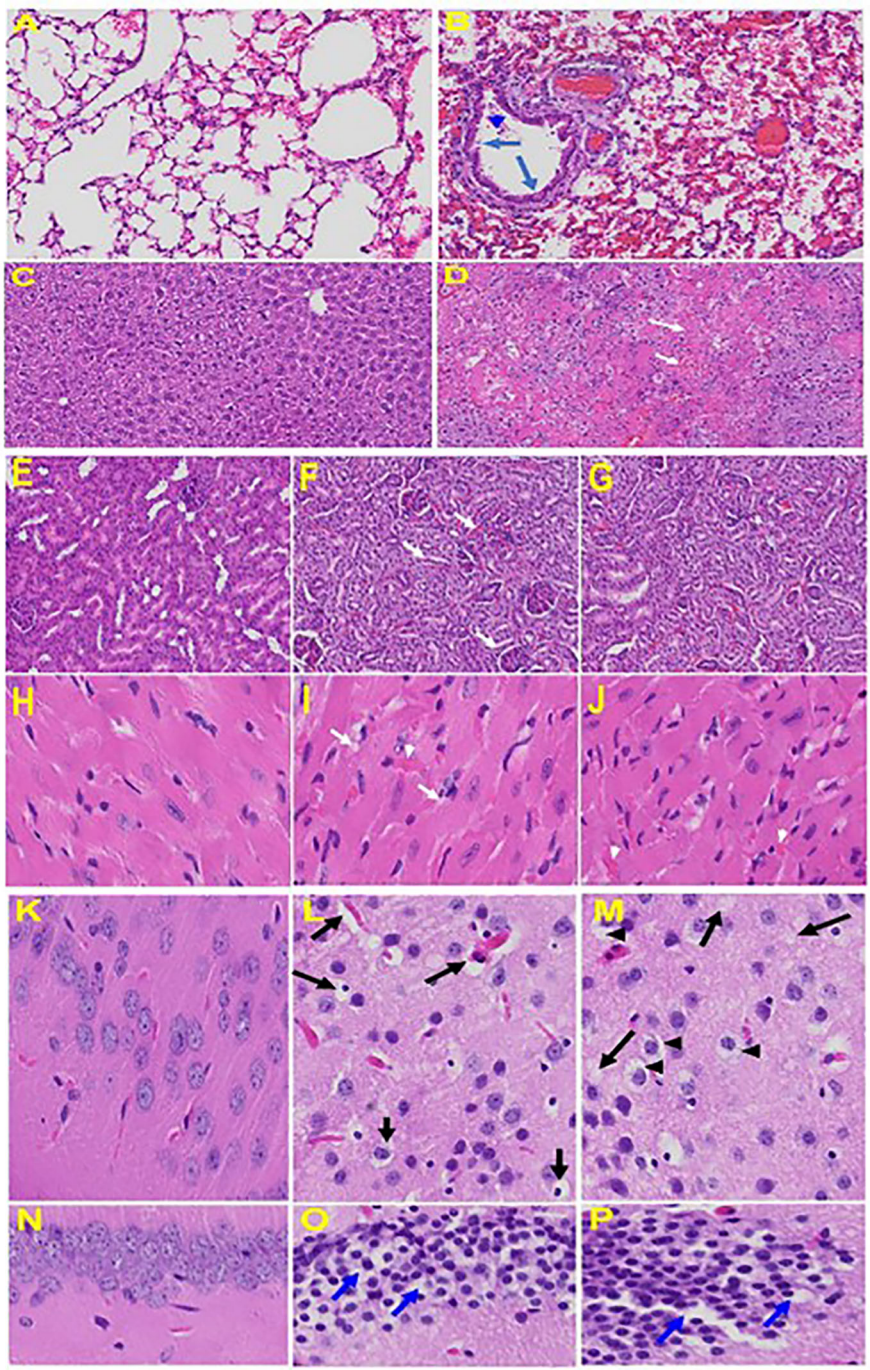
Organs histology in infected and uninfected mice: Representative histological images of haematoxylin and eosin (H&E) stained lung tissue sections of normal mouse **(A)** and infected mouse lung **(B)**. MHV-1-infected mice lung shows arterial endothelial swelling (hypertrophy, long arrow), inflammation and granular degeneration of cells (short arrow) and migration of leukocytes (arrowhead) into lung. Peribronchiolar interstitial infiltration, bronchiole epithelial cell necrosis and necrotic cell debris within alveolar lumens, alveolar exudation, infiltration, hyaline membrane formation and alveolar hemorrhage with red blood cells within the alveolar space and interstitial edema were observed in MHV-1-infected mice. Liver, kidney, heart, and brain tissue degenerative changes were observed in MHV-1-infected mice. MHV-1-infected mice at day 6 showed liver hepatocytes degeneration, severe cells necrosis [**(D)**, long arrows] and hemorrhagic changes (short arrows) when compared to uninfected mice **(C)**; Uninfected kidney **(E)** and tubular epithelial cells degenerative changes and vacuolation, and peritubular vessels congestion **(F, G)**; Severe interstitial edema (arrows), vascular congestion and dilation (arrow heads) and red blood cells infiltrating between degenerative myocardial fibers were seen in the MHV-1 infected mice heart **(I, J)**, as compared to uninfected mice **(H)**. Vacuolation of cerebral matrix [arrows, **(L, M)**] and cytotoxic edema [arrow heads, **(L, M)**], congested blood vessels with perivascular edema [long arrows, **(L, M)**], as well as cytotoxic edema (short arrows) were seen in MHV-1infected mice brain cortex, and in hippocampus (O&P). Reproduced with permission from Paidas et al, Viruses; Published by MDPI, 2021.

Upon examination of the MHV-1 infected mice brain, we observed congested blood vessels, perivascular cavitation (suggestive of edema), pericellular halos, vacuolation of neuropils, darkly stained nuclei and pyknotic nuclei amid associated vacuolation of the neuropil, and acute eosinophilic necrosis (Paidas et al., 2021). MHV-1 infected mice brain hippocampus showed necrotic neuron with fragmented nucleus and vacuolation. The heart of MHV-1 infected mice showed severe interstitial edema, vascular congestion and dilation, and red blood cells infiltrating between degenerative myocardial fibers while tubular epithelial cell degenerative changes, peritubular vessel congestion, proximal and distal tubular necrosis, hemorrhage in interstitial tissue, and vacuolation of renal tubules were observed in MHV-1 exposed mice kidneys, which are identical to that observed in humans associated with SARS-CoV-2 (Paidas et al., 2021). The histopathology observed in this study, as well as the timeline for the progression of clinical symptoms, the high viral load recovered from infected tissues, and the ability to study this model in a Biosafety Level 2 lab further support the use of MHV-1 model in further viral research. These findings collectively suggest that MHV-1 infection in A/J mice is a suitable model to study SARS-CoV-2. See **Figure 6** for organs histology in infected and uninfected mice.

#### 2.5.2 MHV-3

MHV-3 also produces pulmonary lesions, while these changes were milder than those caused by MHV-1 and did not have the characteristic features described in SARS-CoV-2 infection in humans. Additionally, MHV-3-infected mice all developed severe hepatic necrosis and died of liver failure by day 10. Further, A/J mice are resistant to MHV-3 infection, thus MHV-3 did not represent relevant models of SARS-CoV-2.

#### 2.5.3. MHV-A59

Similar to MHV-3, MHV-A59 also produces pulmonary lesions, while these changes were also milder than those caused by MHV-1, and did not have the characteristics of SARS-CoV-2 infection in humans. MHV-A59-infected mice also developed severe hepatic necrosis and died of liver failure by day 10. These findings clearly suggests that both MHV-3 and MHV-A59 strains did not represent relevant models of SARS-CoV-2.

#### 2.5.4. MHV-JHM

MHV-JHM strain, first isolated from a paralyzed mouse in 1949 (Bailey et al., 1949), has been shown to be highly neurotropic, inducing encephalomyelitis and demyelination while weakly affecting liver and lungs cells. De Albuquerque et al. (2006) recently demonstrated how nasal inoculation of BALB/c mice with MHV-JHM failed to produce lung or liver pathology. Other neuropathic effects of neurotropic MHV infection have been studied and include demyelination of CNS leading to overactive bladder (similar to MS), paralysis of lower limbs and muscle wasting (Perlman et al., 1987; Lane et al., 1998; McMillan et al., 2014). MHV-JHM’s increased neuropathogenesis is associated with elevated cytokine levels (IFN-β, IL-1β, IL-6, CCL3, CCL4) (Rempel et al., 2004a). The high neurovirulent lethality of JHM has been associated with a diminished CD8 T-cell immune response to infection (Rempel et al., 2004a). Mice with chronic MHV-JHM infection exhibited astrocyte production of cytokines (TNF-α, IL-1β, and IL-6) in the spinal cord (Sun et al., 1995). MHV-JHM virus has been proven to be recoverable from uterus, placenta and fetus of infected BALB/c mice during all three trimesters of pregnancy (Barthold et al., 1988). MHV-JHM and other highly virulent neurotropic strains, can be considered useful in research regarding the mechanisms of viral entry into the CNS, but due to low pneumotropism, are poor model candidates for COVID19 research.

*MHV-S:* Barthold and Smith (1983) infected 3-week-old mice with MHV-S and analyzed tissues 49 days after inoculation and it was found that 1 week after infection, virus was recovered from brain and lung tissue of most mice and the liver of one mouse. Lesions included olfactory mucosal necrosis, infiltrates and vacuolation of the brain, pulmonary perivascular infiltrates, focal interstitial pneumonia and hepatitis; seroconversion was detected 10 days post-infection and serum titers peaked at 28 days post-infection. While the MHV-S strain seems to have tropism comparable to the MHV-1 strain, which most exhibits the clinical disease progression and histopathology seen in COVID-19 patients, it has been shown by Körner et al. (2020) that MHV-S has low virulence and is detected at low percentages in infected tissue, limiting its usefulness as a COVID-19 model when compared to more virulent MHV strains.

### 2.6 Syrian Hamster Model

**Figure 7:**
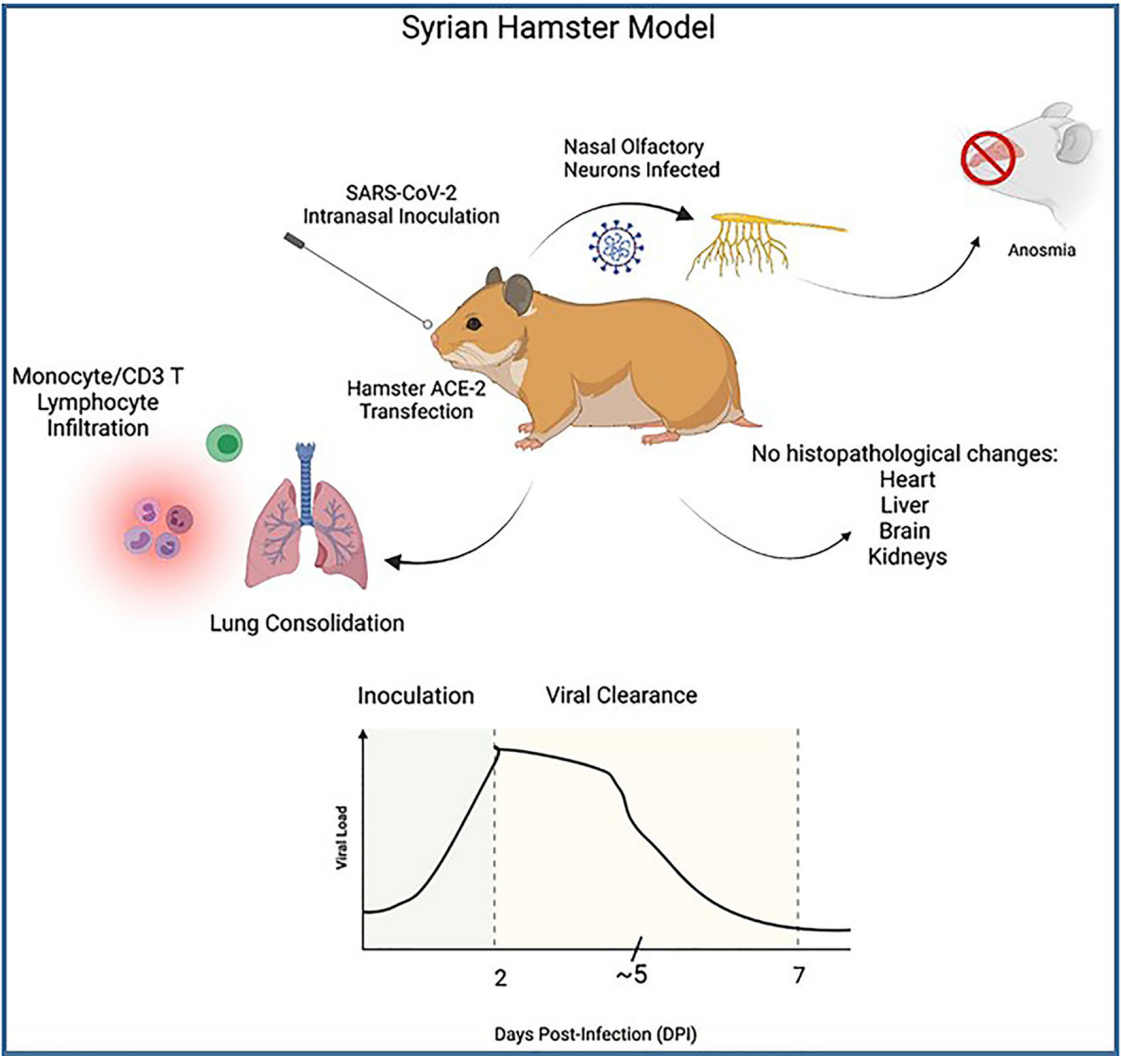
Illustrates the process of Syrian hamster model SARS-CoV-2 inoculation as well as clinical symptoms. Top: Wild-type Hamster ACE-2 are readily susceptible to SARS-CoV-2 infection, particularly nasal olfactory neurons, leading to anosmia. Innate and cell-mediated infiltration were seen at lung with consolidation noted, however no histopathology was seen in extrapulmonary tissues despite viral antigen noted at duodenal and colon epithelia. Bottom: Graph illustrates viral course of SARS-CoV-2 infection in Syrian hamster model. High titers of viral RNA are noted 2 days post-inoculation (DPI), with steady decline of viral load 5 DPI and sharp decline and viral clearance at approximately 7 DPI.

Hamsters, specifically the Golden Syrian Hamster, have been used in SARS-CoV and MERS CoV studies, as well as multiple respiratory infectious diseases including influenza virus. Sia et al. (2020) performed studies to test for pathogenesis of SARS-CoV-2 in 4-5-week-old male Syrian Hamsters. These authors infected the hamsters intranasally 8x10^4^ TCID50 with SARS-CoV-2 virus isolated from cells of a COVID-19 patient from Hong Kong. Sectioned tissue samples were obtained and analyzed for pathology 2-, 5- and 7-days post-infection (DPI) and compared with control studies. The authors of this study concluded that the Syrian hamster was an effective animal model in studying the effects of COVID-19 for several reasons. These include the possibility to observe a consistent progression of illness and clearance of SARS-CoV-2 from 2 to 7 days. Peak viral loads were noted on 2 DPI, detected at bronchial epithelium which then declined 5 DPI (peak viral antigen detected at type II pneumocytes), and was not detectable by 7 DPI. Monocyte infiltration and CD3 T lymphocytes were noted in bronchial epithelium and consolidation changes in the lungs were seen during the course of infection and clearance of the virus. They were also able to observe infection of nasal olfactory neurons which correlate to anosmia in COVID-19 patients (**Figure 7**). Sia et al. were also able to demonstrate transmission of SARS-CoV-2 between inoculated hamsters and naïve hamsters, in a short period after inoculation, which is useful in understanding transmission of SARS-CoV-2 by aerosol droplet and fomites. The fast clearance of the virus in hamsters can also shed light on the immune response mechanism and defense against COVID-19. While there was viral antigen noted in epithelial cells of the duodenum and colon at 2 DPI, which correlates to COVID-19 patients, researchers noted that there were no histopathological changes in samples of inoculated or contact hamster brains, livers, hearts or kidneys. This is a limitation of the hamster model as it would not be useful to study the extrapulmonary pathologies that are observed in patients associated with COVID-19. Yang et al. (2021) recently used the Syrian hamster model to explore biomarkers of COVID-19. Of note, elevated levels of amylase, lipase, GOT/GTP ratio, as well as viral replication in liver and pancreatic tissues were observed in this study suggesting a useful model in the study of the effects of comorbidities on COVID-19 as patients with chronic liver and pancreas disease have been shown to have increased susceptibility to infection and thus face increased risk of mortality to COVID-19.

Notable advantages to the use of hamsters in COVID-19 would be that which is shared with similarly sized models (high reproduction rate, ability to thrive in small housing allows large number of subjects to be studied at a time). Olfactory neurons of the hamster model are readily infected by SARS-CoV-2 which may be useful in studies of COVID-19 patients experiencing lingering anosmia after they have cleared SARS-CoV-2. Disadvantages that were noted based on the studies observed in this review include lack of extrapulmonary manifestations of common COVID-19 that is seen in human patients, and that SARS-CoV-2 is quickly cleared limiting the hamster’s usefulness as a model for severe COVID-19.

### 2.7 Ferret Models

**Figure 8:**
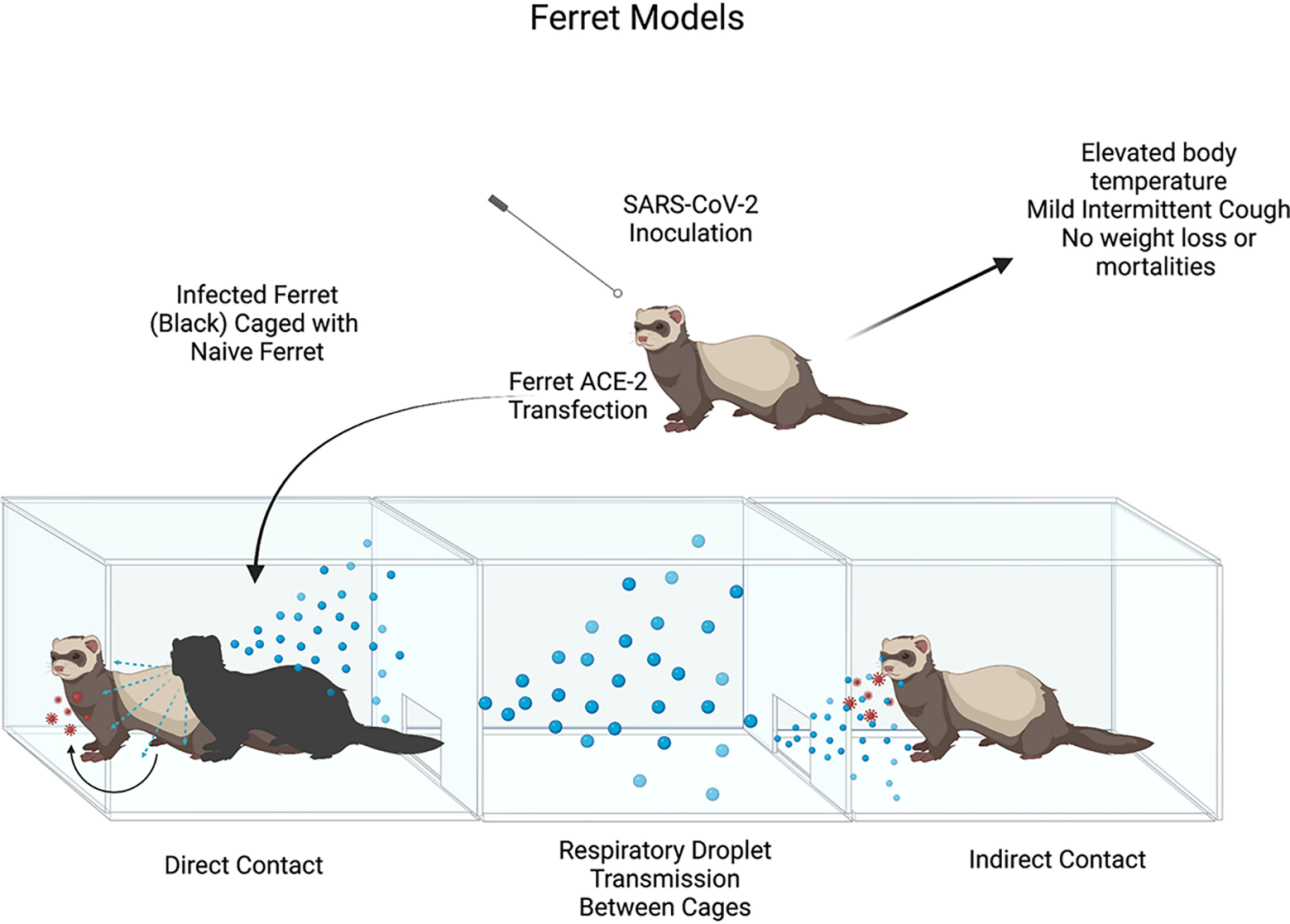
Illustrates the process of Ferret model SARS-CoV-2 inoculation and clinical symptoms post-infection. Illustration of SARS-CoV-2 Ferret Direct vs. Indirect transmission experiment. Ferret ACE-2 receptors are readily susceptible to SARS-CoV-2 infection with mild clinical signs seen in in infected ferrets (hyperthermia, mild cough). High titers of viral loads in upper airways provide evidence for direct & indirect contact leading to viral infection though respiratory droplet transmission.

The ferret model of SARS-CoV-2 infection was first used by Kim et al. (2020). Ferret model was suitable for this goal due to ferret ACE2 having specific SARS-CoV binding proteins, which at the time had been shown to be homologous to SARS-CoV-2 binding proteins. Kim et al. performed 3 trials, inoculating 2 ferrets intranasally with 10^5.5^ TCID50 of NMC-nCoV02 strain from a COVID-19 patient. The study observed different modes of viral infection, direct inoculation vs direct contact vs indirect contact, between the infected ferrets and naïve ferrets. The study noted elevated body temperature changes in all infected ferrets between 2 and 8 DPI, with return to normal body temperature on 8 DPI. Infected ferrets showed mild respiratory symptoms (i.e. occasional cough), no changes in body weight, and no mortalities were observed as had been seen in murine models or humans associated with SARS-CoV-2 infection. It was noted that all direct contact ferrets (which had been housed with directly infected ferrets) experienced elevated body temperature changes 4-6 days post-contact, and no detectable changes in body weight (**Figure 8**).

Benefits of the ferret model would be their natural susceptibility to respiratory viruses such as SARS-CoV-2, a clear advantage compared to transgenic mice models. Another benefit of the ferret model includes the ability to reproduce the human condition of viral infections due to proportional respiratory tract anatomy of the upper and lower airways, as well as the number of terminal bronchioles and density of submucosal glands. Ferrets are also able to produce high titers of viral loads in upper airways and infect naïve ferrets by contact transmission which would be useful in understanding human viral transmission. Limitations of ferret model use would be low viral titers in the lungs and mild clinical symptoms, findings that would not be of much use to understand lung pathology or severe COVID-19 cases. The study did take note that prolonged infection in the lungs at lower viral levels may help in understanding transmission in asymptomatic carriers, a useful model in vaccine experimentation & research.

Corroborating, extensive studies by (Shi et al. 2020) using multiple animal species for supporting infection with SARS-CoV-2 concluded that ferrets were highly susceptible in their upper airways (nasal turbinate, soft palate, and tonsils) but not lung tissue, and may affect the GI tract. Researchers noted more studies were needed to see if male ferrets were more susceptible than females, as is seen in COVID-19 patients.

### 2.8 Non-Human Primate Models

Expansion of SARS-CoV-2 infection studies to non-human primates (NHPs) were explored in an effort to optimize animal models used in COVID-19 research, considering limitations that had been seen in rodents. The benefit of using NHP models is their close physiological relation to the human immune response during infections. This review will focus on studies involved with 3 of the more promising NHP models: Rhesus Macaques, Cynomolgus Macaques and African Green Monkeys.

#### 2.8.1 Rhesus Macaques

Rhesus Macaques were used in the study of Munster et al. (2020) in order to assess their usefulness as animal models in the study of SARS-CoV-2. In this study 8 adult macaques (4 males and 4 females between 4-6 years-old) were inoculated *via* a combination of routes (intranasal, intraocular, intratracheal and oral) with a dilution of the virus. The animals were split into two groups (3 DPI and 21 DPI) and observed for clinical signs. Clinical exams were performed on days 0, 1, 3, 5, 7, 10, 12, 14, 17, 21 and reviewed clinical factors such as bodyweight & temperature as well as chest x-rays. Bronchiolar lavages and histopathological changes from collected tissues were also performed (Munster et al., 2020).

The results of the Munster study showed transient moderate COVID-19 disease as is seen in humans. Of note, pulmonary infiltrates were seen in all test macaques. The rhesus macaque model viral pattern of shedding from upper and lower respiratory tracts was also shown to reflect human viral spread and, similar to humans, there was evidence of viral shedding after resolution of clinical symptoms and radiographic findings. These authors were also able to demonstrate a seroconversion timeframe similar to humans, with IgG antibodies detected 7-10 DPI.

Additional studies by McMahan et al. (2021) using rhesus macaques to understand the immune response to infection suggested a threshold for neutralizing antibody titers which protects against illness in this animal model and the contribution of cell-mediated immunity against infection. This study supports the use of this model in understanding vaccine development and the importance of stimulating humoral immunity in the host.

Further studies by Shaan Lakshmanappa et al. (2021) made use of the rhesus macaque model in their research of CD4 helper T-cell response to SARS-CoV-2 infection. The authors were able to provide evidence of follicular T helper cell generation and Germinal Center stimulation, leading to early production of IgG antibodies. Histopathology lesions in this study showed mild to moderate extensive interstitial pneumonia. However, there were no signs of weight loss, fever, or clinical disease seen in infected macaques and there were no presentations of acute respiratory distress syndrome (ARDS). Benefits of the rhesus macaque model include the displayed high levels of viremia, which can be useful in immunological studies, but due to the lack of clinical disease presentations, limits the model’s usefulness in understanding clinical illness of COVID19 patients.

**Figure 9:**
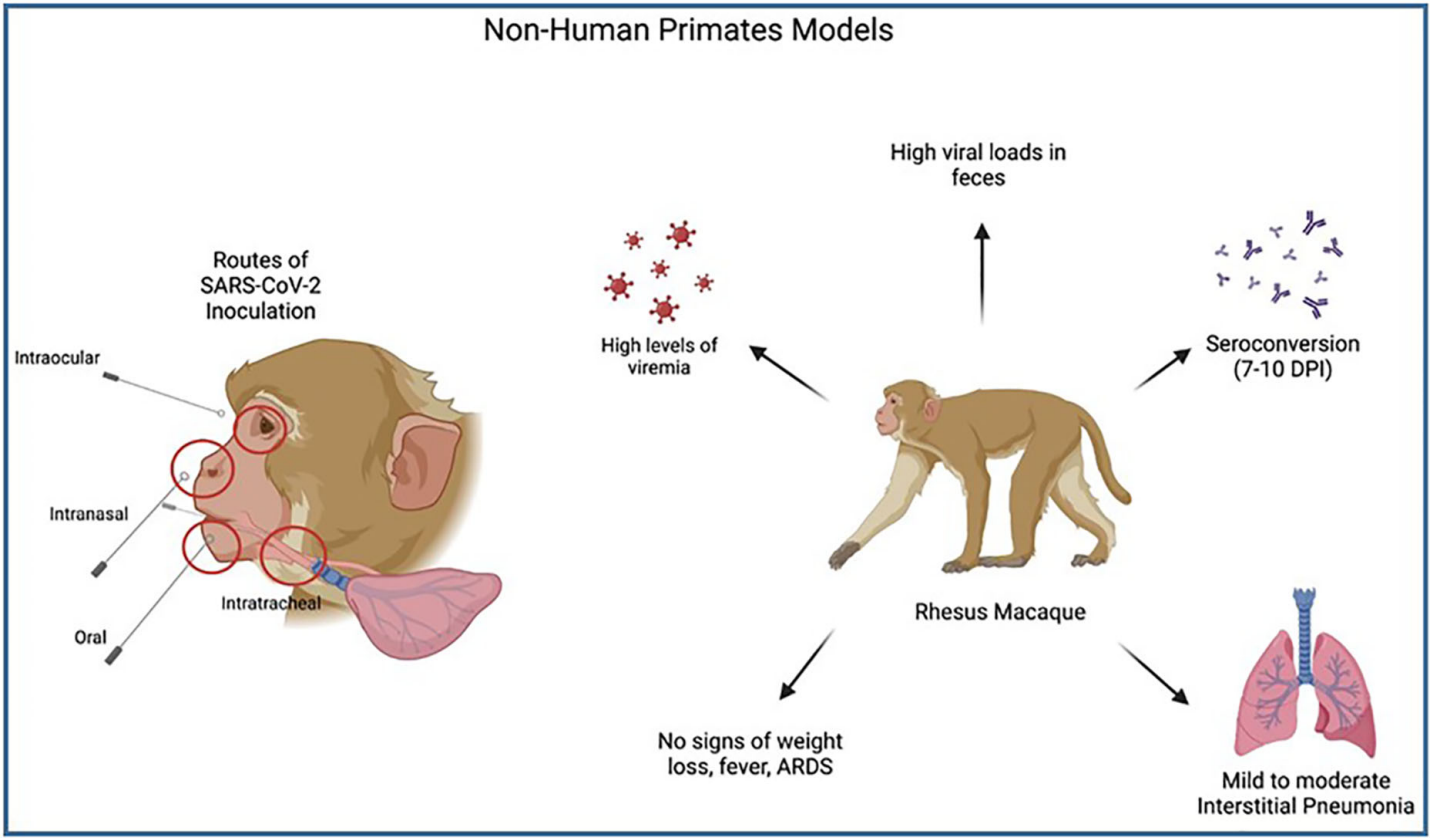
Illustrates the process of Non-Human Primate model SARS-CoV-2 inoculation and clinical symptoms post-infection. Multiple routes of SARS-CoV-2 inoculation allow researchers to understand which area of the upper airways are most susceptible to SARS-CoV-2 infection, with the highest levels found in the pharynx and nasal cavities. A common NHP model, Rhesus macaques, have been shown to have high levels of viremia & viral loads in feces, with seroconversion comparable to humans and mild clinical symptoms (mild-moderate interstitial pneumonia). No systemic symptoms or ARDS were seen in infected macaques making this model less useful in studying severe COVID-19.

Supporting studies by Blair et al. (2021) provided additional evidence for the utilization of rhesus macaques in COVID19 study due to their ability to exhibit mild clinical disease similar to humans. The authors were able to use this model to demonstrate that the highest levels of SARS-CoV-2 viral replication are found in the pharynx and nasal cavity of the host, as is seen in COVID19 patients. The study also showed high viral RNA loads in feces, similar to humans. Multiple routes of infection were used in their rhesus macaque study and it was shown that despite large differences in exposure doses, there were no significant differences in viral RNA loads or kinetics. See **Figure 9**.

#### 2.8.2 Cynomolgus Macaques

The Rockx et al. study (2020) was the first to confirm the basis for the use of cynomolgus macaques as an animal model for SARS-CoV-2 research. This was accomplished by comparing and contrasting histopathology and clinical signs of SARS-CoV-2 with two similar coronaviruses, SARS-CoV and MERS CoV. In their study design, two groups of 4 Cynomolgus Macaques (young adult, 4-5 years- old, and older animals, 15-20 years of age) were inoculated with a SARS-CoV-2 strain by both intratracheal (IT) and intranasal (IN) routes. Only one of the animals showed clinical signs on examination (serous nasal discharge 14 DPI), while no animals exhibited significant weight loss as seen in humans associated with SARS-CoV-2 infection and by 14 DPI all animals had seroconverted antibodies specific to SARS-CoV-2. They were also able to demonstrate viral shedding through mucosal swabbing, RT-qPCR and viral cultures. Of note, aged animals exhibited higher viral levels in nasal swabs, when compared to the younger age group.

Histopathological signs noted at consolidated lung tissue, of both age groups, involved alveoli and bronchiolar luminal tissue which revealed exudative fluid, fibrin, cellular debris and immune cells (mostly alveolar macrophages). As has been seen in other studies, there was evidence of type II pneumocyte hyperplasia with type I pneumocytes being primarily affected.

Researchers concluded that the diffuse alveolar damage (DAD) seen, along with SARS-CoV-2 antigen expression at these lesions, are evidence for the SARS-CoV-2 virus causing the characteristic histopathology that can also be seen in human COVID-19 cases. The data provided in the study showed evidence of cynomolgus macaques usefulness as an animal model for SARS-CoV-2, most specifically their ability to demonstrate upper airway infection & disease transmission and lower airway lung disease. Salguero et al. (2021) further reinforced the usefulness of the cynomolgus macaque model in pathogenesis and histopathological research.

#### 2.8.3 African Green Monkeys

The African Green Monkey (AGM) model, demonstrated by Woolsey et al. (2020), was performed after the success of the cynomolgus and rhesus macaque models demonstrated by the Rockx (2020) and Salguero (2021) studies. AGM was investigated in their immune response to SARS-CoV-2 due to their ability to support the highest levels of replication of SARS-CoV-1 compared to the other NHPs.

In the Woolsey et al. study, the authors inoculated six adult AGMs with 5 x 10^5^ of SARS-CoV-2 isolated from a COVID-19 patient in Italy by both intratracheal and intranasal routes. Three AGMs were euthanized at 5 DPI while the other 3 were observed throughout the experiment (for 21 days). Blood samples were collected from all monkeys on days 0, 2, 3, 4 & 5 and continued on days 7, 9, 12, 15, and 21 for the remaining 3 subjects. The AGMs were analyzed for clinical signs, blood inflammatory markers, immune responses, markers of organ function, chest radiographs and for histopathology.

This study found that there were no signs of notable clinical illness observed in the AGMs, however there were indications of systemic response to infection as was evident by elevated inflammatory markers, leukocytes, and thrombocytes. Elevated temperatures were seen in two animals. All animals held after 5 DPI seroconverted, with two having high IgG titers while one animal held at 21 DPI had low level IgG titers. All AGMs exhibited viral pneumonia and pulmonary consolidation seen on radiographs, as was seen in other NHP studies (Hartman et al., 2020). These findings suggest that factors other than elevated inflammatory markers, leukocytes, and thrombocytes may have been responsible for lung consolidation and it is recommended not to rely solely on chest radiographs for primary diagnosis of COVID-19.

This study also showed evidence of GI involvement in the infected subjects, with all subjects showing abnormalities in the small intestine despite no signs of GI distress. This can be attributed to ACE-2 receptors expressed at the ileum and colon which would also be sites of entry for SARS- CoV-2, which has also been reported in human cases.

Another interesting finding in the data of this study is the similarities in the inflammatory immune response markers in AGMs and humans and the correlation of disease severity and the levels of cytokine markers. Fibrinogen levels were also elevated in most of the infected AGMs, another finding seen in human cases, and may be implicated in thrombosis and ischemic vascular events. A significant advantage of the AGM model is the spectrum of AGM host response to SARS-CoV-2 infection, as is seen in human cases. Such similarities in phenotypic manifestations of COVID-19 in AGMs compared to humans lends support for non-human primates to serve as models in COVID-19 research. Examining COVID-19 biomarkers such as tissue viral load and histopathology in infected AGM tissue can aid in comparing the efficacy of vaccines and therapeutics.

AGMs, and NHPs in general, are more difficult models for use in animal research as they legally have a special status that requires strong justification for their use, they are in need of larger enclosure spaces, must be kept in social groups, have a more varied diet that is more costly to maintain, and need to be habituated to housing & transportation so as to reduce stress levels which can compromise scientific data (Bushmitz, 2014).

#### 2.8.4 Baboons

A recent study compared acute SARS-CoV-2 infection in young and old rhesus macaques and baboons and old marmosets (Singh et al., 2021). Macaques, baboons and old marmosets were infected by multiple routes (ocular, intratracheal and intranasal) with sixth-passage, fully sequenced and authenticated virus at a target dose of 1.05×10^6^ PFU/per animal. SARS-CoV-2 viral RNA (vRNA) was detected early in all species at 3 dpi, and declined thereafter at variable rates. The authors found that baboons had prolonged viral RNA shedding and substantially more lung inflammation compared with macaques. Further, inflammation in bronchoalveolar lavage (BAL) was increased in old versus young baboons. Histopathologic analysis of infected baboons revealed extensive interstitial lymphocytes, plasma cells, lesser macrophages and eosinophils expanding the alveolar septa and alveolar spaces filled with macrophages. Alveolar wall thickening by interstitial deposits of collagen, alveoli lined by occasional type II pneumocytes and alveolar spaces containing syncytial cells and alveolar macrophages were also observed. Using techniques like CT imaging, immunophenotyping, alveolar/peripheral cytokine responses and immunohistochemical analyses, the authors delineated cellular immune responses to SARS-CoV-2 infection in macaque and baboon lungs, including innate and adaptive immune cells and a prominent Type I-interferon response. The authors concluded that acute respiratory distress in baboons recapitulates the progression of COVID-19 in humans, making them suitable as models to test vaccines and therapies.

### 2.9 Cat Model

Studies have utilized cat models in SARS-CoV-2 research due their close proximity with humans and reports of viral human-to-cat transmission. Shi et al. (2020) investigated SARS-CoV-2 replication and respiratory droplet transmission in their 2020 study and provided evidence of respiratory droplet transmission as well as viral replication in inoculated cats. A total of 7 young adult cats (aged 6 to 9 months) were used in the study and intranasally inoculated with 10^5^ PFU of SARS-CoV-2 isolate. In four cats, viral replication in organs was examined after euthanasia on days 3 & 6 post-infection, two cats for each DPI. To study droplet transmission, 3 cats were placed in separate cages with an isolator. An uninfected cat was placed in a cage adjacent to the infected cats’ cages. The authors reported that a limitation of this cat model would be aggressive behavior caused difficulty in collecting regular nasal washings and so feces were collected with viral RNA examined in organs after euthanasia.

It was found that the animals that were euthanized 3 days post-infection had detectable viral RNA in the upper airways (nasal turbinate, soft palate, tonsils, trachea), lower airways (lungs) and small intestines. Both animals euthanized on day 6 post-infection showed no detectable viral RNA in lung samples, while viral RNA was detected in both animals’ nasal turbinates, soft palate and tonsils. The respiratory droplet transmission study showed viral RNA present in feces of two cats 3 DPI and all three cats 5 DPI. Viral RNA was also detected in feces of the exposed cat, providing evidence of feline-to-feline respiratory droplet transmission. The results of this study provided evidence that SARS-CoV-2 is able to replicate efficiently in cats, younger cats were found to be more vulnerable to infection than older cats, and SARS-CoV-2 was found to be transmitted effectively through respiratory droplet transmission, as is seen in COVID-19 patient studies.

Based on the outcomes of the reviewed cat model studies, advantages to this model’s use would be that it is readily susceptible to SARS-CoV-2 infection which is preferable to the use of a transgenic model which would be more costly and time-consuming. Younger animals showed increased susceptibility to infection increases this model’s usefulness in studying mild pediatric COVID19 but limits its functionality as a model for COVID19 in older populations. High titers of viral RNA is readily detected 3 days post-infection which is accelerated relative to other explored models and may lend itself to use in therapeutic research. Notable disadvantages would be difficulty in handling the model itself due to cats known aggressive nature, making recovering i.e. nasal samples difficult. Cats are also at a higher level of regulation in scientific research and require sufficient housing, food, and resources, making the cat model costly for smaller labs to afford.

## 3 *In Vitro* Models of SARS-CoV-2 Infection

*In vitro* cell lines have been used in several studies to aid in the understanding of pathogenesis, tropism and treatment of respiratory virus infections (Rijsbergen et al., 2021). Immortalized cell lines are able to help researchers discover the different cells needed for the infections seen in *in-vivo* models and are able to be manipulated. Through previous SARS-CoV studies performed (i.e. Ren et al., 2006), there has been a clear understanding of the cell lines with the highest concentration of the ACE-2 receptor at their apical side which is needed for SARS-CoV-2 infection. High concentration of ACE-2 receptors was found to correlate with high rates of cell infection. Cell lines such as apical plasma membrane of polarized respiratory epithelial cells, colon carcinoma cell line (Caco-2) (Pascoal et al., 2021), a lung carcinoma cell line (Calu-3) (Kanimozhi et al., 2021) and Vero E6 cells (Zupin et al., 2021) have been put forward as candidates for models in COVID19 research. Human cancer cell lines were screened for the SARS-CoV-2 cellular entry factors ACE2 and TMPRSS2 based on RNA-seq data of the Cancer Cell Line Encyclopedia (CCLE). These findings suggest that surface expression of ACE2 in polarized epithelial cells function as receptor for severe acute respiratory syndrome.

*Vero E6:* Vero E6 cell lines, extracted from kidney epithelial cells of African Green Monkeys, are used in the testing of various agents and drugs. Due to the fact that these cells are obtained from normal epithelial cells, and not immortal cell lines, they retain many normal cell functions and are used regularly in viral studies, most notably recent and on-going SARS-CoV-2 research (Prieto, 2002). The usefulness of Vero E6 in SARS-CoV-2 studies is highlighted by this cell line’s high ACE2 receptor expression which cause this cell line to be vulnerable to SARS-CoV-2 infection, which can be enhanced by engineering Vero E6 to express the TMPRSS2 protein which is needed for S protein priming (Matsuyama et al., 2020). This model was also used to aid in a SARS-CoV-2 pathogenesis study in which the accumulation of lipids was seen in infected *in vitro* Vero E6 cells as well as lungs of COVID19 patients, suggesting that lipids are involved in SARS-CoV-2 pathogenesis (Nardacci et al., 2021). Vero E6 cells were recently used in a study determining the *in vitro* minimal exposure time and viral concentration needed to establish persistent SARS-CoV-2 infection (Zupin et al., 2021). Vero E6 cell lines have also been used in multiple studies of SARS-CoV-2 repurposed antivirals (Canal et al., 2021, Leneva et al., 2021), protease inhibitors (Cui and Jia, 2021; Han et al., 2021) as well as inhibiting peptides (Kelesidis et al., 2021). This cell line model was used in research investigating the indirect inhibition of the ACE-2 receptor as a possible SARS-CoV-2 therapeutic (Tanimoto et al., 2021) along with human polyclonal IgG (Gilliland et al., 2021). Treating SARS-CoV-2 infection with repurposed drugs has been an area of intense research due to the speed at which these therapeutics can be approved and implemented into the clinical setting. Vero E6 cell lines were used in the investigations of Losartan (Nejat et al., 2021), Doxycycline (Gendrot et al., 2020), and Fluoxetine (Dechaumes et al., 2021). Scientists have cautioned the use of Vero E6 cell lines in serial propagation of SARS-CoV-2 due its ability to rapidly generate virus variants relative to other cell lines (Funnell et al., 2021).

*Caco-2:* Caco-2 cell lines, an immortal cell line derived from human colorectal adenocarcinoma cells, are actively used in many areas of research, particularly in gastrointestinal studies such as the Pascoal et al. study (2021) which used the Caco-2 model to conclude that changes in microbiota and short-chain fatty acids produced do not interfere with SARS-CoV-2 intestinal infection. Caco-2 cells have been particularly useful in SARS-CoV-2 research due to the fact that unlike Vero E6, which also expresses ACE-2, Caco-2 is able to express the TMPRSS2 coreceptor endogenously, unlike Vero E6 in which TMPRSS2 expression must be induced (Lee et al., 2020). Caco-2 cell line has shown to be useful in areas of research investigating the nature of SARS-CoV-2 such as comparing & contrasting the kinetics of variants *in vitro* (Touret et al., 2021), the role of integrins in SARS-CoV-2 pathogenesis (Nader et al., 2021), and SARS-CoV-2 transcriptional & post-transcriptional processing dynamics in infected cells (Chang et al., 2021). Similar to Vero E6 cell lines, Caco-2 was also used in studies investigating 6 repurposed drugs in the treatment of SARS-CoV-2 infection with activity against SARS-CoV-2 (i.e. Amiodarone, Lactoferrin, Remdesivir) (Mirabelli et al., 2021). The use of miRNA targeting the SARS-CoV-2 TMPRSS2 coreceptor to prevent cell entry has also been studied using the Caco-2 cell line. (Kaur et al., 2021)

Huh-7: Huh-7, derived from human colorectal adenocarcinoma, is another popular cell line model that has had uses in multiple areas of viral and drug research. The Huh-7 cell line was found to also exhibit ACE-2 receptor protein expression (Kaye, 2006) and so it has been selected in several SARS-CoV-2 studies including investigations of anti-HCV drugs against SARS-CoV-2 (Sacramento et al., 2021), repurposing of viral inhibitors (Puhl et al., 2021), testing of an immunomodulating herbal extract (Roshdy et al., 2020) and traditional Chinese medicine effect on signaling pathways involved in SARS-CoV-2 pathogenesis (Ma et al., 2020).

*A549*: A549, derived from Human lung adenocarcinoma, is a cell line that similar to previously discussed cell lines, is sensitive to SARS-CoV-2 infection and has been made useful in SARS-CoV-2 investigations. A549 has been most recently used in studies concerning viral replication cycle kinetics (Bernhauerová et al., 2021), SARS-CoV-2 accessory proteins (Silvas et al., 2021), Ebola and Marburg Virus Inhibitors (Puhl et al., 2021), infection- induced promoter hypomethylation (Muhammad et al., 2021), SARS-CoV-2 protease 3CL^pro^ inhibitors (de Vries et al., 2021), and 7 FDA-approved antivirals (Weston et al., 2020).

There were shared similar advantages and disadvantages between the use of cell lines when compared to the use of animal models. Cell lines were found to be cost-effective in that there is no required housing of cages, food and low lab maintenance costs. Experiments are also easier to perform and immortal cell lines have the benefit of an unlimited supply as well as bypassing the ethical and legal drawbacks of animal model use. Disadvantages that ultimately limit the usefulness of cell line as a model for COVID19 research include the problem of results of *in-vitro* studies not being reproducible in *in-vivo* studies due to the complex interplay of multiple physiological processes that include multiple (i.e. inflammatory cytokines, signaling molecules, hormone dynamic changes). Genetically modified tissues may also not behave similarly to *in-vivo* tissues and so *in-vitro* has been relegated to use in understanding protein-receptor relationships.

In the Wurtz et al. study (2021), 34 cell lines derived from both animal and human tissues were investigated for supportive infection of SARS-CoV-2. While this study proposed the use of various cell lines for SARS-CoV-2, Vero E6, Caco-2, Huh-7, Calu-3, and A549 cell lines are more representative due to similar factors. Refer to **Table 1** for cell line data.

**Table 1:** Cell lines susceptible to SARS-CoV-2 infection.

No.	Cell line	ACE-2 (Presence or Absence)	Effectiveness	Reference
1	Vero E6	Presence	Yes	(Cui and Jia (2021); Dechaumes et al., (2021); Funnell et al., (2021); Gendrot et al., (2020); Gilliland et al., (2021); Han et al., (2021); Leneva et al., (2021); Kelesidis et al., (2021); Matsuyama et al., (2020); Nardacci et al., (2021); Nejat et al., (2021); Prieto et al., (2002); Tanimoto et al., (2021); Wurtz et al., (2021); Zupin et al., (2021))
2	Caco-2	Presence	Yes	(Chang et al., (2021); Kaur et al., (2021); Lee et al., (2020); Mirabelli et al., (2021); Nader et al., (2021); Pascoal et al., (2021); Touret et al., (2021); Wurtz et al., (2021)
3	Huh-7	Presence	Yes	(Kaye et al., (2006); Ma et al., (2020); Puhl et al., (2020); Roshdy et al., (2020); Sacramento et al., (2021); Wurtz et al., (2021))
4	Calu-3	Presence	Yes	(Kanimozhi et al., (2021); Wurtz et al., (2021))
5	A549	Absence	No	(Bernhauerová et al., (2021); de Vries et al, (2021); Muhammad et al., (2021); Puhl et al., (2021); Silvas et al, (2021); Weston et al., (2020); Wurtz et al., (2021))
6	HEK-293T	Presence	No	Wurtz et al., (2021)
7	LNCaP	Absence	No	Wurtz et al., (2021)
8	Aa23	Absence	No	Wurtz et al., (2021)
9	C6/36	Absence	No	Wurtz et al., (2021)
10	S2	Absence	No	Wurtz et al., (2021)
11	ISE6	Absence	No	Wurtz et al., (2021)
12	IPL-LD-65Y	Absence	No	Wurtz et al., (2021)
13	BGM	Presence	Yes	Wurtz et al., (2021)
14	VERO/hSLAM	Presence	Yes	Wurtz et al., (2021)
15	MA104	Presence	Yes	Wurtz et al., (2021)
16	VERO81	Absence	No	Wurtz et al., (2021)
17	LLC-MK2	Presence	Yes	Wurtz et al., (2021)
18	HT-29	Absence	No	Wurtz et al., (2021)
19	HELA	Absence	No	Wurtz et al., (2021)
20	HCT-8	Absence	No	Wurtz et al., (2021)
21	HEP-2	Absence	No	Wurtz et al., (2021)
22	ECV304	Absence	No	Wurtz et al., (2021)
23	HL-60	Absence	No	Wurtz et al., (2021)
24	MRC5	Absence	No	Wurtz et al., (2021)
25	THP1	Absence	No	Wurtz et al., (2021)
26	BHK21	Absence	No	Wurtz et al., (2021)
27	McCoy	Absence	No	Wurtz et al., (2021)
28	L929	Absence	No	Wurtz et al., (2021)
29	P388D1	Absence	No	Wurtz et al., (2021)
30	RAW 264.7	Absence	No	Wurtz et al., (2021)
31	BA 886	Absence	No	Wurtz et al., (2021)
32	MDCK	Absence	No	Wurtz et al., (2021)
33	DH82	Absence	No	Wurtz et al., (2021)
34	OA3.Ts	Absence	No	Wurtz et al., (2021)
35	MDOK	Absence	No	Wurtz et al., (2021)
36	R05T	Absence	No	Wurtz et al., (2021)
37	R06E	Absence	No	Wurtz et al., (2021)
38	TB1 Lu	Absence	No	Wurtz et al., (2021)
39	XTC-2	Absence	No	Wurtz et al., (2021)

ACE-2 presence increases both susceptibility to infection and cell line’s effectiveness in use for COVID-19 research and has been shown to correlate with the presence of ACE-2 receptors in cell line tissue. The cell lines that have been found to be effective, and most useful in COVID-19 research include VeroE6, Caco-2, A549, Huh-7, & Calu-3.

## 4 Conclusion

Reliable animal models are important in the development of vaccines and therapeutics. Optimizing the animal model in the study and research of a specific pathogen can streamline research efforts towards vaccines and cures. SARS-CoV-2 vaccine & therapeutic development was accelerated due to the worldwide effort to share resources and data. *In-vitro* cell lines aided in the understanding of the interaction between the human ACE-2 receptor and SARS-CoV-2 spike protein which was first discovered in prior SARS-CoV research. Using this foundational research and understanding of coronavirus pathogenesis, *in-vivo* research would be the next step in combating COVID-19 and animal models would need to be thoroughly investigated for optimal use in therapeutic and vaccine exploration. Of the animal models discussed in this review, there were many benefits and limitations to consider depending on the goal of the intended research of each study Refer to **Table 2** for summarized conclusions of the review conclusions of the review. Most models were able to demonstrate transmission, pathogenesis, and histopathology similar to human cases. There were nuances to be explored within each animal model that would shed light on COVID-19. Infection with SARS-CoV-2 in hamsters demonstrated demographic differences in response as seen in humans (males and elderly experience more severe illness vs females and young). Studies in hamsters can be completed quickly and in a cost-effective manner. Ferrets were shown to be readily infected without the need for transgenics (such as mice species), however infection was mostly restricted to upper respiratory passages, which would not lend this model to lung pathology research. Ferret models would be useful in research into viral shedding from nasal and oropharyngeal epithelium and asymptomatic transmission in COVID-19 patients. Transgenic mice were shown to be useful in the understanding of mild extrapulmonary disease, infectious encephalitis, thrombosis and anosmia. Adv-hACE2 mice were shown to be the most useful murine model due to their ability to quickly infect any and all lab mice, regardless of genetic predisposition to infection.

**Table 2:** Experimental models of SARS-CoV-2 with characteristic features, advantages and disadvantages.

Experimental Model	Characteristics	Advantages	Disadvantages	References
Non-Human Primates				
Cynomolgus Macaques	Histopathological signs noted at consolidated lung tissue involved alveoli and bronchiolar luminal tissue which revealed exudative fluid, fibrin, cellular debris and immune cells (mostly alveolar macrophages). Aged animals exhibited higher viral levels at nasal swabs, when compared to the younger age group	• Physiologically closest in similarity to humans • Timeline of COVID19 clinical symptoms and seroconversion similar to humans Aged Rhesus macaques & Baboons highly susceptible to COVID19, useful for studying older age and SARS-coV-2 infection AGMs good model for inflammatory biomarkers of COVID19	• Detailed regulation for housing NHPs requires sufficient housing, food, and resources – extremely costly and most difficult model to maintain • Slow reproduction rate • AGMs poor model for clinical COVID19 and other NHP models lack any clinical presentation of disease despite high viremia	(Rockx et al., 2020; Salguero et al., 2021)
Rhesus Macaques	Seroconversion timeframe similar to humans, with IgG antibodies detected 7-10 DPI. Highest levels of SARS-CoV-2 viral replication are found in the pharynx and nasal cavity of the host			(Blair et al., 2021; McMahan et al., 2021; Munster et al., 2020; Shaan et al., 2021)
African Green Monkeys	Viral pneumonia and pulmonary consolidation seen on radiographs of all subjects. No signs of notable clinical symptoms. Elevated inflammatory markers, leukocytes, fibrinogen and thrombocytes. Elevated temperatures seen in few subjects.			(Bushmitz et al., 2013; Hartman et al., 2020; Rockx et al., 2020; Salguero et al., 2021; Woolsey et al., 2020)
Baboons	Prolonged viral RNA shedding and substantial lung inflammation. Extensive interstitial lymphocytes, plasma cells, lesser macrophages and eosinophils expanding the alveolar septa and alveolar spaces filled with macrophages. Alveolar wall thickening by interstitial deposits of collagen, alveoli lined by occasional type II pneumocytes and alveolar spaces containing syncytial cells and alveolar macrophage.			(Singh et al., 2021)
Ferret	Respiratory tract anatomy of the upper and lower airways proportional to humans, as well as the number of terminal bronchioles and density of submucosal glands. Produce high titers of viral loads in upper airways	• Direct contact transmission significant • Do not require spacious housing due to small size • Highly susceptible to SARS-CoV-2 infection • Prolonged infection at lower levels in lungs may be useful in understanding asymptomatic COVID19	• Low viral titers in the lungs and mild clinical symptoms, poor model for severe COVID19	(Kim et al., 2020; Shi et al., 2020)
Syrian Hamster	• Monocyte infiltration and CD3 T lymphocytes noted in bronchial epithelium • Consolidation changes in the lungs seen during the course of infection and clearance of the virus. Able to observe infection of nasal olfactory neurons which correlate to anosmia in COVID-19 patients	• Able to reproduce quickly Direct contact transmission significant Do not require spacious housing due to small size Infection of nasal olfactory neurons may be useful in COVID-19 patients exhibiting anosmia post-infection	• Virus is quickly cleared, not a good model for severe COVID19 • Do not manifest common extrapulmonary symptoms seen in humans	(Sia et al., 2020; Yang et al., 2021)
**Mouse Models**				
K18-hACE2 Transgenic Mice	Epithelial cell cytokeratin-18 promoter (k-18) hACE2 gene	• All mouse models able to reproduce quickly • Do not require spacious housing due to small size • K18-hACE2 model useful for mild extrapulmonary clinical COVID (i.e. anosmia) Adv-hACE2 model able to infect mice readily without trangenesis MHV model able to be studied in BSL-2 lab	• Producing transgenic mice can be costly • Most mouse models are not able to produce severe clinical symptoms seen in COVID19 • Some transgenic mouse models (k18-hACE2, mACE2-hACE2) may have ectopic hACE2 expression limiting their usefulness	(Arce and Costoya, 2021; Ejaz et al., 2020; Liu et al., 2021; Seibert et al., 2021; Winkler et al., 2020; Zheng et al., 2021)
mACE2-hACE2 Transgenic Mice	Mouse ACE-2 promoter with hACE-2 coding sequence			(Bao et al., 2020)
Endogenous mACE2 Transgenic Mice	Sequential passaging of SARS-CoV-2 virus over time causes an increase in viral tropism for the mouse ACE-2 receptor			(Huang et al., 2021; Zhang et al., 2021)
Adv-hACE2 Transgenic Mice	Transduction infection of mouse with Adenovirus with hACE-2 gene			(Goh et al., 2020; Hartman et al., 2020; Qiu et al., 2020)
Mouse Hepatitis Virus Model*	MHV virus (Coronavirus family) produces identical SARS-CoV-2/COVID19 tissue pathology and clinical symptoms compared to humans			(Bailey et al., 1949; Barthold and Smith, 1983; Davidson et al., 1987; Barthold et al., 1988; Rempel et al., 2004a; Kim et al., 2020; McMahan et al., 2021; Nejat et al., 2021; Rees-Spear et al., 2021; Singh et al., 2021)
Cat	Feline-to-feline respiratory droplet transmission & viral replication	• Wild-types susceptible to SARS-CoV-2 infection Younger animals more vulnerable to infection Viral RNA detectable 3 DPI	• Aggressive behavior caused difficulty in collecting regular nasal washings – feces collected Detailed regulation for housing cats requires sufficient housing, food, and resources - costly	(Shi et al., 2020)
***In Vitro* Cell Lines**				
Vero E6	Kidney epithelial cells of African Green Monkey	• Cost effective compared to housing of animal models • Easy to use • Unlimited supply	• Genetically modified tissues may not reproduce physiological *in-vivo* state Limited uses for clinical research Results do not take into account complex physiological interplay seen in *in-vivo*	(Cui and Jia, 2021; Dechaumes et al., 2021; Funnell et al., 2021; Gendrot et al., 2020; Gilliland et al., 2021; Han et al., 2021; Leneva et al., 2021; Kelesidis et al., 2021; Matsuyama et al., 2020; Nardacci et al., 2021; Nejat et al., 2021; Prieto et al., 2002; Tanimoto et al., 2021; Wurtz et al., 2021; Zupin et al., 2021)
Caco-2	Human colorectal adenocarcinoma cells			(Chang et al., 2021; Kaur et al., 2021; Lee et al., 2020; Mirabelli et al., 2021; Nader et al., 2021; Pascoal et al., 2021; Touret et al., 2021; Wurtz et al., 2021)
Huh-7	Human hepatocellular carcinoma			(Kaye et al., 2006; Ma et al., 2020; Puhl et al., 2020; Roshdy et al., 2020; Sacramento et al., 2021; Wurtz et al., 2021)
A549	Human lung adenocarcinoma			(de Vries et al., 2021; Bernhauerová et al., 2021; Muhammad et al., 2021; Puhl et al., 2021; Silvas et al., 2021; Weston et al., 2020; Wurtz et al., 2021)

NHP models were ultimately shown to be the most representative of COVID-19 disease process as seen in humans. Baboons have been shown in studies to support SARS-CoV-2 replication and may be useful in understanding the effects of comorbidities, such as cardiovascular disease, and their interactions with the virus. Macaques and African green monkeys were shown to support a high level of viral replication at low viral load and display a similar spectrum of clinical disease as is seen in human patients, allowing research to study mild to severe illness and work towards therapeutics to combat these effects of infection, yet NHP models are an under-utilized model in research due to their high costs.

Of the models investigated in this study, it is the suggestion of this review that the Murine Hepatitis Virus-1 model be considered an excellent and most optimal research model in future COVID19 studies. The MHV-1 virus model is able to replicate the life cycle of a beta coronavirus at physiological doses with analogous histopathology of infected tissues as well as represent the broad spectrum of clinical disease seen in COVID19 patients, lending this model to further our understanding of this illness. The MHV-1 model is also able to replicate viral transmission seen in humans and has similar pneumotropism as well as extrapulmonary effects seen in human patients. Immunological host response and seroconversion is proportional to that seen in human studies and most importantly the MHV-1 model is able to be used at low cost, is highly practical, and most importantly can be studied in a Biosafety Level-2 lab setting, and provides acceptable ecological, as well as ethical consequences during experimentation. Balancing safety, mimicking human COVID-19 and robustness of the animal model, the Murine Hepatitis Virus-1 model currently represents the most suitable model for SARS-CoV-2/COVID19 research.

## Author Contributions

LC-C, MP, and AJ contributed to conception and design of the review. All authors contributed to the writing of the manuscript.

## Conflict of Interest

The authors declare that the research was conducted in the absence of any commercial or financial relationships that could be construed as a potential conflict of interest.

## Publisher’s Note

All claims expressed in this article are solely those of the authors and do not necessarily represent those of their affiliated organizations, or those of the publisher, the editors and the reviewers. Any product that may be evaluated in this article, or claim that may be made by its manufacturer, is not guaranteed or endorsed by the publisher.
